# Origin and Evolution of the Multifaceted Adherens Junction Component Plekha7

**DOI:** 10.3389/fcell.2022.856975

**Published:** 2022-03-23

**Authors:** Antonis Kourtidis, Bryan Dighera, Alyssa Risner, Rob Hackemack, Nikolas Nikolaidis

**Affiliations:** ^1^ Department of Regenerative Medicine and Cell Biology, Medical University of South Carolina, Charleston, SC, United States; ^2^ Department of Biological Science, Center for Applied Biotechnology Studies, Center for Computational and Applied Mathematics, College of Natural Sciences and Mathematics, California State University, Fullerton, Fullerton, CA, United States

**Keywords:** Plekha7, Plekha6, Plekha5, Plekha4, PLEKHA, phylogeny, chordates, gnathostomes

## Abstract

Plekha7 is a key adherens junction component involved in numerous functions in mammalian cells. Plekha7 is the most studied member of the PLEKHA protein family, which includes eight members with diverse functions. However, the evolutionary history of Plekha7 remains unexplored. Here, we outline the phylogeny and identify the origins of this gene and its paralogs. We show that Plekha7, together with Plekha4, Plekha5, and Plekha6, belong to a subfamily that we name PLEKHA4/5/6/7. This subfamily is distinct from the other Plekha proteins, which form two additional separate subfamilies, namely PLEKHA1/2 and PLEKHA3/8. Sequence, phylogenetic, exon-intron organization, and syntenic analyses reveal that the PLEKHA4/5/6/7 subfamily is represented by a single gene in invertebrates, which remained single in the last common ancestor of all chordates and underwent gene duplications distinctly in jawless and jawed vertebrates. In the latter species, a first round of gene duplications gave rise to the Plekha4/7 and Plekha5/6 pairs and a second round to the four extant members of the subfamily. These observations are consistent with the 1R/2R hypothesis of vertebrate genome evolution. Plekha7 and Plekha5 also exist in two copies in ray-finned fishes, due to the Teleostei-specific whole genome duplication. Similarities between the vertebrate Plekha4/5/6/7 members and non-chordate sequences are restricted to their N-terminal PH domains, whereas similarities across the remaining protein molecule are only sporadically found among few invertebrate species and are limited to the coiled-coil and extreme C-terminal ends. The vertebrate Plekha4/5/6/7 proteins contain extensive intrinsically disordered domains, which are topologically and structurally conserved in all chordates, but not in non-chordate invertebrates. In summary, our study sheds light on the origins and evolution of Plekha7 and the PLEKHA4/5/6/7 subfamily and unveils new critical information suitable for future functional studies of this still understudied group of proteins.

## Introduction

The appearance of elaborate structures allowing cells to adhere to each other and compose tissues and organs enabled the emergence of animal multicellularity. The adherens junction (AJ) is a major cell-cell adhesion protein complex present throughout animals and is required for body morphogenesis, tissue formation and maintenance, and cellular signaling (Harris and Tepass, 2010; Takeichi, 2014; Kourtidis et al., 2017a; Daulagala et al., 2019). The core component of the AJ is a classical cadherin protein family member, such as E-cadherin, which associates intracellularly with p120 catenin (p120), β-catenin, and α-catenin (Harris and Tepass, 2010; Kourtidis et al., 2013; Takeichi, 2014). Evolutionarily, cadherin-like molecules first appeared in choanoflagellates, the closest living relatives of multicellular animals (Brunet and King, 2017). However, classical cadherins with cell-cell adhesion properties and catenin-binding domains that form AJs, appeared later in animal evolution, in Placozoans and Cnidarians. Classical cadherins have diversified from one or two members in invertebrates into a large protein family that includes 32 members in vertebrates (Oda and Takeichi, 2011; Oda, 2012; Gul et al., 2017). α- and β-catenin homologs have also been identified in non-metazoans, such as *Dictyostelium discoideum*, and are present in all metazoans, either as a single member in invertebrates, or as two to three members in vertebrates (Hulpiau et al., 2013; Gul et al., 2017). Similarly, p120 has evolved from a single protein in invertebrates (δ-catenin) to seven proteins in vertebrates (Anastasiadis and Reynolds, 2000; Ireton et al., 2002; Fang et al., 2004; Carnahan et al., 2010). Collectively, these findings underscore the increased complexity of the AJs in vertebrates.

In addition to the essential cadherin-catenin composition, numerous other proteins participate in AJ formation, playing critical roles in their structure and function. An AJ protein that has gained attention in recent years is PLEKHA7 (pleckstrin homology domain containing, family A, member 7). PLEKHA7 was originally identified as an E-cadherin–p120 binding partner, specific to the apical AJs in mature epithelial tissues and monolayers, tethering the minus ends of the microtubules to the AJs (Meng et al., 2008; Pulimeno et al., 2010; Pulimeno et al., 2011). Since then, multiple additional roles have been attributed to PLEKHA7, including cell-cell junction stabilization through interactions with junctional and cytoskeletal components (Kurita et al., 2013; Paschoud et al., 2014; Guerrera et al., 2016; Kourtidis and Anastasiadis, 2016; Lee et al., 2017); recruitment and regulation of the RNA interference (RNAi) machinery at AJs (Kourtidis et al., 2015; Kourtidis et al., 2017b; Nair-Menon et al., 2020); regulation of hypertension (Endres et al., 2014); function as a tumor suppressor (Kourtidis et al., 2015; Tille et al., 2015; Rea et al., 2018; Nair-Menon et al., 2020; Pence et al., 2021); regulation of responses to bacterial toxins (Popov et al., 2015; Shah et al., 2018), and involvement in neocortex development (Tavano et al., 2018). Although these studies exemplify the importance of PLEKHA7 in human health and disease, little is known regarding the roles of PLEKHA7 in non-mammalian species. The only exception is the zebrafish PLEKHA7 homolog, Hadp1, which has been implicated in cardiac contractility during embryogenesis (Wythe et al., 2011).

**FIGURE 1:**
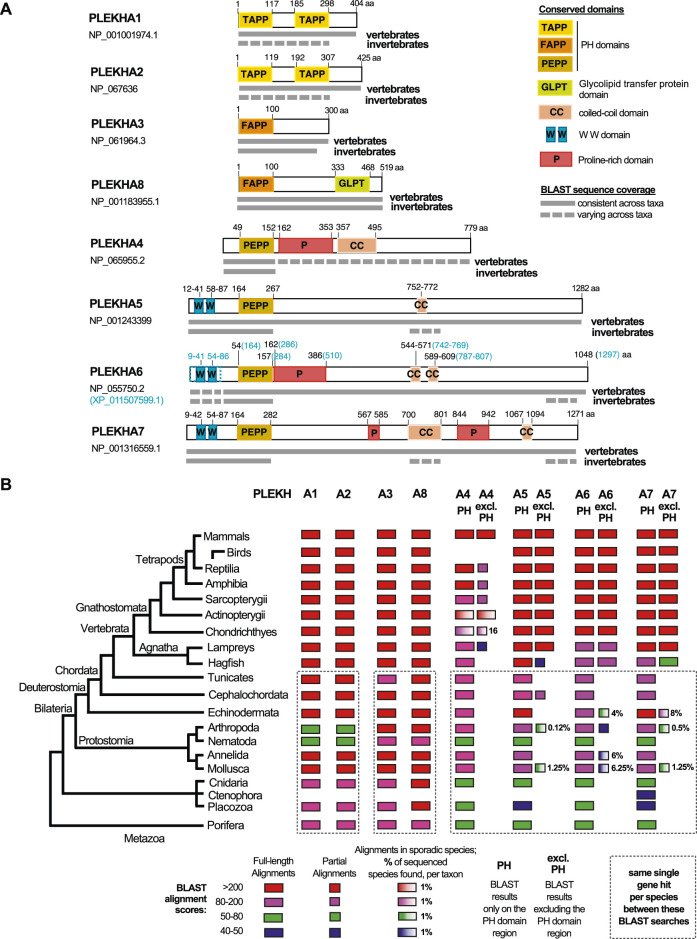
Comprehensive summary of the BLAST searches conducted in the present study. **(A)** Graphical summaries of the sequence coverage of the human PLEKHA1-8 protein sequences in metazoan genomes, as retrieved through the BLAST searches. Grey boxes depict sequence coverage of alignments in vertebrate and invertebrate proteins, juxtaposed to their amino acid (aa) position. Colored boxes show the domain organization of the human proteins. See also key panel on the top right. Accession numbers under each PLEKHA name indicate the sequences used for the BLAST searches; a predicted isoform of PLEKHA6 (XP_011507599.1) that includes the documented WW domains of this protein is also outlined, since the single NCBI-verified PLEKHA6 isoform (NP_055750.2) lacks these domains. **(B)** Diagram showing sequence identities of the human PLEKHA1-8 proteins in metazoan genomes, as retrieved through the BLAST searches. BLAST results are shown separately for the PH domain region only and for the region excluding the PH domain (excl.PH) for the PLEKHA4,5,6,7 members. Color and box-width codes are shown at the bottom of the Figure. The numbers on the right side of the boxes represent the percentage of genomes in which sporadic alignments were identified, as opposed to all the currently sequenced genomes per taxon. BLAST searches of different human PLEKHA sequences that retrieved the same single gene per species, are enclosed in dotted boxes. Metazoan taxa are presented on the left on a standard consensus phylogenetic tree. See also Supplementary Tables S1–S10 and tabs therein for the complete sets of BLAST results.

The emergence of PLEKHA7 as a critical component of the AJs with multifaceted roles in mammalian cells and models and the overall lack of information regarding this protein in other taxa, prompted us to investigate PLEKHA7’s evolutionary history and potential origin, which are currently unknown. PLEKHA7 belongs to the PLEKHA protein family that includes eight members, namely PLEKHA1, PLEKHA2, PLEKHA3, PLEKHA4, PLEKHA5, PLEKHA6, PLEKHA7, and PLEKHA8, due to all containing pleckstrin homology (PH) domains (Dowler et al., 2000). Considering that these proteins have distinct domain organization (Dowler et al., 2000) (Figure 1A) it remains unclear whether they are paralogs and share a common evolutionary history, or just share a common evolutionary domain. Furthermore, their PH domains possess distinct phosphoinositide binding properties and belong to functionally different PH domain families (Dowler et al., 2000; Sluysmans et al., 2021b), whereas only PLEKHA4, PLEKHA5, and PLEKHA6 potentially associate with cadherin complexes, in addition to PLEKHA7 (Marshall et al., 2002; Godi et al., 2004; Balla et al., 2005; Shami Shah et al., 2019; Aleshin et al., 2021; Sluysmans et al., 2021a; Sluysmans et al., 2021b; Troyanovsky et al., 2021). Overall, database entries (e.g., NCBI; GeneCards - www.genecards.org) (Stelzer et al., 2016) provide inconclusive descriptions of the paralogous relationships of the PLEKHA family. Thus, we hereby sought to clarify the phylogenetic relationships between PLEKHA7 and the other PLEKHA family members and to reconstruct their evolutionary history, using molecular evolution, sequence, and bioinformatics analyses.

## Methods

### Sequence Collection, Multiple Sequence Alignment, and Domain Prediction Analysis

To determine the evolutionary pathway and conservation of PLEKHA7 and its domains, Plekha sequences were collected from the National Center for Biotechnology Information (NCBI) reference protein (RefSeq) database (as of December 2021) using protein-BLAST. Since most Plekha proteins exhibit multiple alternatively spliced variants, we used as queries the longest sequence-verified protein isoform (Supplementary Tables S1A,B). The BLAST searches were performed using default parameters and individually for the major taxonomic databases to ensure the collection of all available proteins. Specifically, mammals were searched separately, followed by birds, ray-finned fishes (Actinopterygii), reptiles, amphibia, cartilaginous fish (Chondrichthyes), and all other vertebrate and invertebrate taxa with available genome sequences at NCBI (Supplementary Table S1A). Additionally, we used the ENSEMBL database to search for Hagfish genes (Genome assembly: Eburgeri_3.2; GCA_900186335.2). Because mammals, birds, and ray-finned fishes have disproportionally more species’ genomes sequenced compared to other taxa, we also narrowed our search in a set of representative species, when collecting sequences for phylogenetic tree construction (Supplementary Table S1A).

These BLAST searches retrieved several homologous sequences, including multiple isoforms, verified or putative, across most species (Supplementary Tables S1B, S2–S9). From this original sequence pool in subsequent analyses, we again retained the largest verified (or predicted) Plekha isoform to avoid redundancy. Overall, protein sequences with alignments producing the lowest E-values (denoting nonrandom alignment between query and target sequences), query coverage greater than 60%, and similar domain organization as the query proteins, were collected. Additionally, to identify common ancestors of these genes in other Chordates or invertebrates, sequences with lower coverage, often limited to the PH domain or to the non-PH-containing regions, were also collected and further analyzed.

The selected sequences were then aligned with the Multiple Alignment using Fast Fourier Transform (MAFFT) program version 7 (Katoh et al., 2002), using the E-INS-i strategy with a BLOSUM62 scoring matrix, a gap opening penalty of 1.53, and a 0.0 offset value. Alignments were also in part performed using the SnapGene software (v5.2.1) (from Insightful Science; available at snapgene.com). Domain identification was performed using the conserved domains database (CDD) (Marchler-Bauer et al., 2015) and published work. Multiple sequence alignments were used to generate sequence conservation logos with the WebLogo software (Crooks et al., 2004). Intrinsically Disordered Region (IDR) predictions were performed at the PSIPRED server (McGuffin et al., 2000; Buchan and Jones, 2019) using the DISOPRED3 function (Jones and Cozzetto, 2015).

### Phylogeny Reconstruction

To determine the phylogenetic relationships of the collected sequences, we generated Neighbor-Joining (NJ) and Maximum likelihood (ML) phylogenetic trees. After analyzing and verifying all sequences’ relationships, different protein sequences were selected in the final dataset as representative sequences (species) based on whether they represented complete sequences and their alignment did not introduce more than 20% of the total gaps.

Maximum-likelihood (ML) was used to find the best model of evolution (MEGA X) (Kumar et al., 2018). Based on the Bayesian Information Criterion (BIC) the substitution pattern was best described by the JTT model with corrections for non-uniform evolutionary rates among sites (+G) and by assuming that a certain fraction of sites is evolutionarily invariable (+I). Phylogenetic trees were generated using the NJ and ML algorithms as implemented in MEGA X. For ML, initial tree(s) for the heuristic search were obtained automatically by applying Neighbor-Join and BioNJ algorithms to a matrix of pairwise distances estimated using the JTT model, and then selecting the topology with superior log likelihood value. In both cases, one thousand bootstrap pseudo-replicates were used to test the reliability of the inferred trees. Phylogenetic trees were constructed using a midpoint root and without the use of other outgroups, as previously proposed for families with highly divergent outgroups, which is the case for the PLEKHA family (Wheeler, 1990; Foster and Hickey, 1999; Huelsenbeck et al., 2002; Shavit et al., 2007; Rota-Stabelli and Telford, 2008; Rosenfeld et al., 2012).

### Analysis of Introns and Genomic Organization

To verify orthology and support homology between the selected sequences, intron position and phase conservation were assessed. Both analyses were performed using *in-house* scripts assembled to a pipeline (www.github.com/bdighera/orthotracer). The intron position and phase for each collected *Plekha* gene was determined by using Spidey to align genomic sequences and coding sequences collected from GenBank (Wheelan et al., 2001). Raw sequence data were collected *via* the RefSeq nuccore database API (application programming interface) using biopython version 3.79 Entrez functions. Then, the introns were mapped on a protein multiple sequence alignment (MSA) to illustrate their position along the molecules. The intron phase (0, no codon split by the intron; 1, codon split by intron at the first codon position; 2, codon split by intron at the second codon position) was calculated by the modulo of exon lengths and denoted with different colors.

To determine whether the synteny observed between *Plekha* and its neighbor genes in humans is conserved in other species, the first four genes on either side, excluding RNA coding genes, and up to 500 kbps from the parent *Plekha* gene/locus were collected, mapped, and analyzed using the RefSeq genomic information. Other established technology leveraged in the *in-house* scripts included Clustal 1.2.1 (Sievers et al., 2011) to generate the MSA using default parameters, and the ete3 package for rendering the genomic synteny and intron phase visualizations (Huerta-Cepas et al., 2016; Sievers and Higgins, 2018; Sievers et al., 2020). Final figures were generated using Adobe Illustrator 25.2 and Affinity Designer 1.10.

### Protein Structural Predictions and Analysis

Protein structural predictions were collected from the AlphaFold Protein Structure Database (Jumper et al., 2021). Figures containing individual three-dimensional models or superimposed structural models were generated using Pymol ™ 1.7.4.5 (Delano Scientific). The models were drawn in cartoon representations and structural alignments were performed using the function “super” in pymol, which executes a sequence independent structure-based dynamic programming alignment between structural elements.

## Results

### Distinct Paralogous Subgroups Exist Within the PLEKHA Family

To investigate the evolution and phylogenetic position of Plekha7, we performed BLAST searches using the human PLEKHA sequences as queries against the NCBI protein database for all major metazoan taxonomic groups (Figures 1A,B; Supplementary Table S1A **)**. In these searches, we considered low E-values (ranging from 0 to 10^−20^) and query coverage >60% as criteria suggesting full-length homology and potential orthology.

Based on these criteria, we consistently identified full-length homologous sequences in all available vertebrate and most invertebrate genomes for the Plekha1, 2, 3, 8 proteins (Figures 1A,B; Supplementary Tables S2–S5). However, the Plekha4, 5, 6, 7 proteins showed consistent full-length homologies only in vertebrates (Figures 1A,B; Supplementary Tables S6–S9). More specifically, searches using the human Plekha4, 5, 6, 7 sequences as queries identified putative orthologs in almost all vertebrate classes examined, including the jawless vertebrate lampreys and hagfish (Figures 1A,B **;** Supplementary Tables S6–S9). BLAST searches failed to identify putative Plekha4 orthologs in birds, and in multiple Actinopterygii species (Figure 1B; Supplementary Table S6). Overall, Plekha4 exhibited lower sequence conservation among vertebrates, compared to Plekha5, Plekha6, or Plekha7 (Figures 1A,B **;** Supplementary Table S6). Notably, the functionally documented PLEKHA6 region coding for the WW domain, (Sluysmans et al., 2021b), was missing from many Plekha6 hits from different species, possibly due to isoform specificity or incomplete annotation (Figure 1A; Supplementary Table S8).

Contrary to vertebrates, BLAST searches using the Plekha4, 5, 6, 7 sequences as queries against invertebrate genomes, resulted in hits with relatively high E-values and low coverage. Most of these alignments were restricted to the N-terminal region containing the PH domain with a few exceptions (Figures 1A,B **;** Supplementary Tables S6–S9–see related taxa-specific tabs). These exceptions included sporadic alignments restricted to the coiled-coiled domains of Plekha7 and Plekha5 or to the extreme C-terminus region of Plekha7, Plekha6 in a few evolutionarily unrelated invertebrates. Similar results were also obtained when we excluded the PH domain and repeated the BLAST searches against invertebrate genomes (Figures 1A,B **;** Supplementary Tables S6–S9–see related taxa-specific tabs).

To further investigate the lack of full-length homologs in invertebrates, we performed BLAST searches using a protein sequence (XP_035672282.1) of the cephalochordate *Branchiostoma floridae* (invertebrate, chordate), as the closest living relative of both vertebrates and non-chordate invertebrates. These searches produced alignments with relatively higher identity scores and from more species in non-chordate invertebrate genomes, compared to the human Plekha4,5,6,7 searches, but were still overall sporadic and limited to the C-terminus (Supplementary Table S10).

These results prompted us to also examine a *Drosophila* gene named *kramer*, which has been reported as a homolog of PLEKHA4 (Shami Shah et al., 2019). BLAST searches using the human PLEKHA4 against the insect and *Drosophila* databases (Supplementary Table S6; see related tabs) showed that the homology between PLEKHA4 and kramer, or other dipteran sequences, was restricted to the PH domain. BLAST searches using kramer as query to search chordate databases retrieved Plekha7 sequences as top hits, as well as several Plekha6, Plekha5, and Plekha4 hits. Reciprocal BLAST searches using the human PLEKHA5,6,7 sequences to query *Drosophila* databases also retrieved kramer as the top hit (Supplementary Tables S6–S9; Supplementary Table S11). Collectively, these results suggest that *kramer* is not a *Plekha4* ortholog, rather the protein contains a PH domain that is closest to the PH domain of all Plekha4,5,6,7 vertebrate proteins.

Overall, searches using Plekha1 retrieved Plekha2 sequences (and vice versa) and they both retrieved the same single gene in invertebrate species (Figure 1B; Supplementary Tables S2,3). Similarly, Plekha3 searches retrieved Plekha8 sequences (and vice versa) and both retrieved the same single member in each invertebrate species (Figure 1B; Supplementary Tables S4,5). Furthermore, each of the Plekha4, Plekha5, Plekha6, Plekha7 BLAST searches retrieved sequences from the other three members in vertebrates, and all four retrieved only one single sequence per species in invertebrates (Figure 1B; Supplementary Tables S6–S9 **)**. Additionally, we did not retrieve any alignments between Plekha1,2 and Plekha3,8, or between any of those and Plekha4,5,6,7, either in vertebrates or in invertebrates (Figure 1B; Supplementary Tables S2–S9). Taken together, the BLAST results suggest that there are at least three distinct sets of genes within the PLEKHA family, the *Plekha1/2*, *Plekha3/8*, and *Plekha4/5/6/7* and that PLEKHA might not be a single family. To further determine the validity of these patterns we performed phylogenetic analysis using the retrieved Plekha proteins.

### The PLEKHA Family Consists of Three Distinct Subfamilies in Metazoans

**FIGURE 2:**
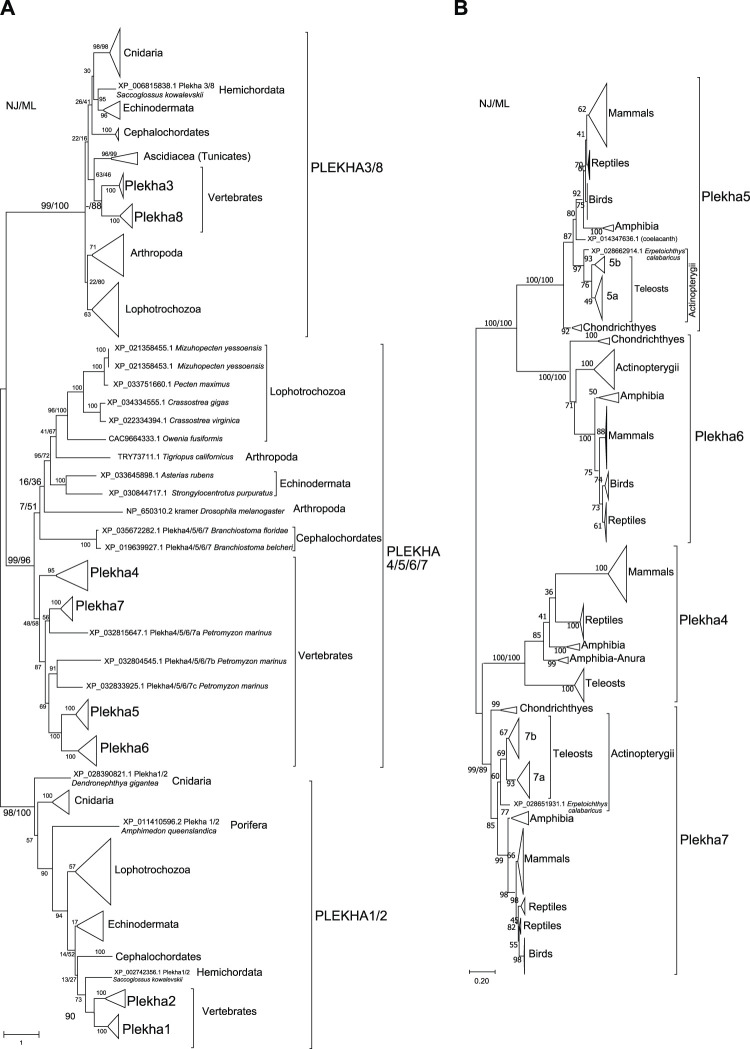
Phylogenetic analyses of the PLEKHA protein family reveal the existence of distinct subfamilies and paralogous groups. **(A)** Evolutionary relationships of all the members of the PLEKHA protein family, as inferred by the Neighbor-Joining and the Maximum Likelihood methods using JTT matrix-based model. The optimal tree and the tree with the highest log likelihood (-123550.77) are shown. A discrete Gamma distribution was used to model evolutionary rate differences among sites [5 categories (+*G*, parameter = 2.0629)]. The tree is drawn to scale, with branch lengths measured in the number of substitutions per site. This analysis involved 107 amino acid sequences. All positions containing gaps and missing data were eliminated (complete deletion option) and a total of 77 positions were used (see Supplementary Figure S1 for uncompressed tree). **(B)** Evolutionary relationships of the vertebrate Plekha4,5,6,7 proteins, as inferred using the Neighbor-Joining and the Maximum Likelihood methods based on the JTT matrix-based model, with complete deletion of amino acid positions containing gaps. There was a total of 245 positions in the final dataset. The optimal tree and the tree with the highest log likelihood (-16497.96) are shown. A discrete Gamma distribution was used to model evolutionary rate differences among sites [5 categories (+*G*, parameter = 1.5563)]. The tree is drawn to scale, with branch lengths measured in the number of substitutions per site. This analysis involved 231 amino acid sequences. The major clades are compressed for better visualization (see Supplementary Figure S2 for uncompressed tree). In both figures, bootstrap values (numbers at nodes for NJ and ML) indicate the percentage of 1,000 replicates in which the associated taxa clustered in the resulting tree (values from both trees are shown only for major clades). A dash instead of a bootstrap value indicates that the branch was not supported by a specific method. Teleost-specific copies are indicated by 5a,b and 7a,b; lamprey-specific copies are indicated by 4/5/6/7a,b,c.

To investigate the phylogenetic relationships within the PLEKHA family, we generated multiple sequence alignments and constructed phylogenetic trees using Plekha sequences from representative species from all Metazoans retrieved from our BLAST searches (Figure 1B; Supplementary Tables S1–S10). Because *Plekha1/2* and *Plekha3/8* orthologs were identified throughout metazoa, we included representative species from both vertebrates and invertebrates. In the case of the Plekha4/5/6/7 sequences, we included representative vertebrates and those invertebrate sequences that showed homologies in both the PH and non-PH regions (Figure 1B; Supplementary Tables S6–S10). These phylogenetic analyses clearly separated the PLEKHA family into three distinct clades: the Plekha4/5/6/7, the Plekha1/2, and the Plekha3/8 clades (Figure 2A; Supplementary Figure S1 **)**. However, confidence was overall low in several internal nodes, since comparisons were based on the PH domain sequence, the only homologous region between all the members of the PLEKHA family.

Within the Plekha1/2 clade, Plekha1 and Plekha2 formed distinct groups within vertebrates, while all invertebrate species studied contained a single member, misannotated in NCBI as either 1 or 2 (Figure 1B, Figure 2A; Supplementary Figure S1). Furthermore, the Plekha1 and Plekha2 sequences follow the species’ phylogeny and were found in almost all species searched (Figure 1B, Figure 2A, Supplementary Figure S1). Together, these findings suggest that the vertebrate *Plekha1* and *Plekha2* genes emerged after the duplication of a single *Plekha1/2* gene that preexisted in invertebrates.

Within the Plekha3/8 clade, Plekha3 and Plekha8 form two distinct groups in vertebrates while invertebrates contain only a single member, misannotated as either Plekha3 or Plekha8 (Figure 1B, Figure 2A; Supplementary Figure S1). The invertebrate Plekha3/8 branching pattern differs from the established species phylogeny although the topology is supported by very low bootstrap scores. Still, results within the Plekha3/8 clade support the idea of a gene duplication event in the last common ancestor of vertebrates that gave rise to *Plekha3* and *Plekha8* from an ancestral single invertebrate *Plekha3/8* gene.

Within the Plekha4/5/6/7 clade, the vertebrate sequences grouped separately from the invertebrate ones (Figure 2A; Supplementary Figure S1 **)**. Within the vertebrate Plekha4/5/6/7 clade, each of the jawed vertebrate (gnathostome) Plekha4, Plekha5, Plekha6 and Plekha7 sequences formed distinct clades (Figure 2A; Supplementary Figure S1 **)**. However, the three Plekha4/5/6/7 sequences from the jawless vertebrate (agnathan) *Petromyzon marinus* didn’t follow the same pattern: one of them formed an outgroup to the jawed vertebrate Plekha7 clade and two formed a distinct clade as an outgroup to the jawed vertebrate Plekha5-Plekha6 clade. Notably, in both cases, clustering of the *Petromyzon* sequences within the jawed vertebrate clades was with relatively low bootstrap values and probably resulted in poor bootstrap values for some of the jawed vertebrate Plekha4,5,6,7 clades (e.g., Plekha4). To resolve the Plekha4/5/6/7 relationships within jawed vertebrates with higher confidence, we excluded the invertebrate and *Petromyzon* sequences and reconstructed the phylogenetic tree (Figure 2B). Indeed, this approach resolved the jawed vertebrate Plekha4,5,6,7 relationships and provided topologies supported by higher bootstrap values. More specifically, the jawed vertebrate Plekha5 and Plekha6 grouped together, whereas Plekha7 and Plekha4 formed a separate clade. The Plekha5/6 clade exhibits the largest distance from the root, with Plekha5 and Plekha6 clades exhibiting similar distances after their split, suggesting a pattern of divergent evolution (Figure 2B; Supplementary Figure S2). Differently, the Plekha4/7 clade exhibits the shortest branch distance from the root with the Plekha4 and Plekha7 showing different divergence patterns (Figure 2B; Supplementary Figure S2). This phylogenetic pattern is consistent in all jawed vertebrate taxa examined, indicating that putative gene duplications may have occurred in the last common ancestor of jawed vertebrates (Figure 2B; Supplementary Figure S2).

The supposition that the *Plekha4/5/6/7* genes are products of duplications in jawed vertebrates is supported by the fact that sequences of the jawless vertebrate *Petromyzon marinus*, were all consistently placed outside the jawed vertebrate gene-specific clades (Figure 2A; Supplementary Figure S1). This topology predicts that the lamprey sequences might represent lineage specific duplications and that the common vertebrate ancestor contained a single *Plekha4/5/6/7* gene. To further evaluate this prediction, we performed phylogenetic analyses including the single Plekha4/5/6/7 sequence (XP_035672282.1; Plekha5-like) from the cephalochordate *Branchiostoma floridae*, which we considered a proper outgroup for two reasons. First, this sequence was retrieved in all Plekha4,5,6,7 BLAST searches; and second, cephalochordates are considered the closest living relative of the common ancestor of all chordates. Indeed, when we included this sequence, it branched out as an outgroup to all Plekha4,5,6,7 members, including the *Petromyzon* sequences (Supplementary Figure S3). A potential interpretation of these results is that a single ancestral *Plekha4/5/6/7* gene existed in the last common ancestor of all chordates and then multiplied and diversified in the present-day members of the family in vertebrates. Corroborating this notion, all four PLEKHA4,5,6,7 BLAST searches also identified a single Plekha4/5/6/7 sequence in the Tunicates *Phallusia mammillata* (CAB3264938.1) and *Ciona robusta* (*Ciona intestinalis* XP_026694274.1) (Supplementary Tables S6–S9). However, we didn’t include these sequences in subsequent analyses, due to either exhibiting poor similarity or being partially annotated.

Phylogenies of the vertebrate Plekha4,5,6,7 proteins overall follow the established species phylogenies (Figure 2B; Supplementary Figure S2, Supplementary Figures S4–S7). Notably, Plekha7 and Plekha5 Actinopterygii orthologs separate into two subgroups (Figure 2B; Supplementary Figures S4,6), each including two distinct copies of each gene (*Plekha7a* and *Plekha7b*; *Plekha5* and *Plekha5-like*; renamed as *5a* and *5b*). Of the two *Plekha7* copies in zebrafish, *Plekha7a* is the copy previously referred to as *Hadp1* (Wythe et al., 2011) (NP_001129715.1; Supplementary Figure S4). Differently, the Actinopterygii *Plekha6* and *Plekha4* orthologs seem to exist in only one copy (Supplementary Figures S5,7). Plekha4 is also missing from many Actinopterygii species examined, suggesting secondary loss of this gene in these species, similarly to its loss in all birds (Figure 1B; Supplementary Figure S7).

Consistent with the idea of a single *Plekha4/5/6/7* gene in chordates, the non-chordate sequences used in our analyses formed outgroups to the chordate clade including the *Drosophila* kramer (Figure 2A). In some taxonomic groups, e.g., Lophotrochozoa or Eleutherozoa, the invertebrate Plekha4/5/6/7 members follow the species phylogeny, and these branches are supported by high bootstrap values. However, in most other cases the branching pattern was supported by very low bootstrap values (Figure 2A). This issue is most probably due to the short homologies, restricted in the PH domains, and due to the fact that these sequences were sporadically found in invertebrates and represent phylogenetically very divergent species (Figure 1B; Supplementary Tables S6–S10).

Taken together, our phylogenetic analyses revealed the presence of three distinct PLEKHA subfamilies in Metazoans, namely PLEKHA1/2, PLEKHA3/8, and PLEKHA4/5/6/7. Additionally, these analyses outline an evolutionary pattern, where the individual members within each of the three subfamilies emerged in vertebrates from a single ancestral invertebrate member. However, this analysis could not fully reconstruct the evolutionary history of the whole Plekha4/5/6/7 protein sequence, which contains an extensive non-PH region that is present in all vertebrates but absent from most invertebrates.

### The Vertebrate Plekha4/5/6/7 Gene Cluster Originates in Early Chordate Evolution

To substantiate the above-described evolutionary patterns and clarify the origins of the *Plekha4/5/6/7* vertebrate genes, we sought to investigate their exon-intron organization and syntenic relationships, as performed previously for other gene families (Nikolaidis and Nei, 2004; D'Aniello et al., 2008; Coppola et al., 2019; Nguyen et al., 2020).

**FIGURE 3:**
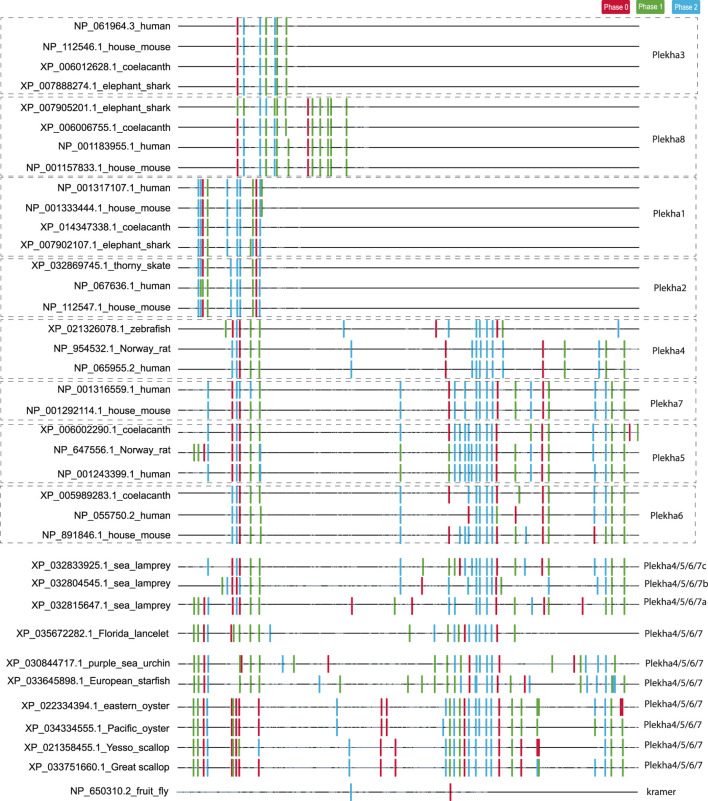
Analysis of intron position and phase confirms the existence of three distinct PLEKHA subfamilies and reveals similarities and differences between vertebrate and invertebrate *Plekha4/5/6/7* genes. The figure depicts the position and phase of introns within *Plekha* genes from representative species. Intron positions were mapped on a multiple sequence alignment. The introns are denoted with colored boxes (phase 0: red; phase 1: green; phase 2: blue). The parts of the multiple sequence alignment that contain information (amino acids) are shown as black lines and the ones containing alignment gaps are shown are light grey lines. The intron collection, mapping, and coloring were performed using *in-house* scripts (see Methods).

Exon-intron organization analysis showed that the three major clades identified in our phylogenetic analyses (*Plekha4/5/6/7*, *Plekha1/2*, and *Plekha3/8*; Figure 2A) have different intron positions and phase (Figure 3). These differences are apparent even within the N-terminus, which includes the PH domain, the only common denominator between all Plekha members (Figure 1A), further underlying the distinct origin and evolution of these three phylogenetic clades.

In contrast, intron position and phase are highly conserved within each phylogenetic clade (Figure 3). In the case of the *Plekha1/2* and *Plekha3/8* genes, this conservation extends in almost all animal species studied. In the case of the *Plekha4/5/6/7* genes, most of the introns were conserved between and within the specific gene clades across vertebrates (Figure 3; Supplementary Figure S8). This conservation (in both the N- and C-termini) is also apparent in the lamprey *Petromyzon marinus* genes (Figure 3; Supplementary Figure S8). Furthermore, the vertebrate *Plekha4/5/6/7* exon-intron organization is also conserved in both the N-terminus and partially in the C-terminus in the single *Plekha4/5/6/7* gene from the cephalochordate *Branchiostoma floridae*. Although many intron positions are also conserved in the other invertebrate sequences analyzed, the phase of these introns is quite different, suggesting changes in the open reading frame of the resulting proteins (Figure 3; Supplementary Figure S8). These partially conserved intron positions in the C-terminus of the invertebrate genes coincide with the coiled-coil domain and the extreme C-terminal domain sequences identified in our BLAST searches (Figure 1A). These findings support the prediction that a single ancestral gene that contained a conserved set of introns, gave rise to the vertebrate *Plekha4/5/6/7* clade. In contrast, exon-intron organization of *kramer* was entirely different from any of the *Plekha* genes examined, further indicating its high diversification and ambiguous origin (Figure 3; Supplementary Figure S8).

Syntenic analysis showed that the *Plekha4/5/6/7* genes share no common genomic neighborhood with the remaining of the PLEKHA family. Instead, the other two subgroups of *Plekha* paralogs exhibit syntenic relationships: the *Plekha1*–*Plekha2* paralogs with *Tacc2*–*Tacc1* and *Htra1*–*Htra4* paralogs and the *Plekha3*–*Plekha8* with the *Fkbp7*–*Fkbp14* (Supplementary Figure S9). Together with our phylogenetic analyses above, these data support the idea of a separate origin and evolution of the PLEKHA4/5/6/7 subfamily from the PLEKHA1/2 and the PLEKHA3/8 ones. Some of the *Plekha4/5/6/7* genes are located on well-studied paralogons (Lagman et al., 2013; Ocampo Daza and Larhammar, 2018), one of which corresponds to paralogon “D” in the reconstruction of the ancestral chordate genome based on the amphioxus genome (Putnam et al., 2008). Similarly, the chromosomal regions around the *Plekha3/8* genes correspond to the “C” ancestral chromosome, and the regions around the *Plekha1/2* genes correspond to the “E” ancestral chromosome (Putnam et al., 2008).

**FIGURE 4:**
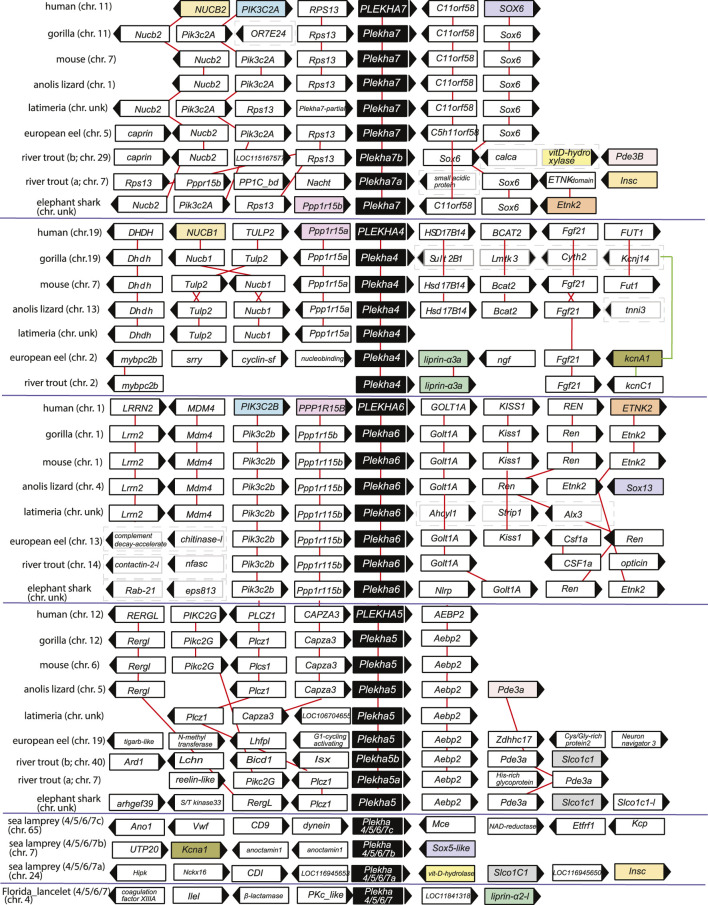
The genomic organization of the *Plekha4,5,6,7* loci is relatively conserved within chordates and reveals ancient genomic duplication events. The first four protein coding genes and up to 500 kbps flanking representative *Plekha4,5,6,7* loci were collected from GenBank using *in-house* scripts (see Methods and Supplementary Figure S10 for complete dataset). The gene name and domain information for each locus were collected and analyzed to suggest homology and determine conserved synteny. Pairwise alignments were used as needed to supplement the above analyses. Genes are represented as arrows and each arrow’s direction represents the gene’s transcription orientation. Information of the chromosome (chr.) is also provided if known as a number or with the abbreviation unk (if unknown). Red lines connect orthologous genes. Genes from humans or fishes are represented as colored boxes only if they were shared at least between two paralogous gene groups. Cases of loss of synteny are represented with dashed boxes around the genes. Teleost-specific copies are indicated by a, b; lamprey-specific copies are indicated by 4/5/6/7a,b,c. The graph is not in scale for clarity.

Syntenic analyses and comparisons between the neighboring genomic regions of the four *Plekha4/5/6/7* genes further solidified orthology between the different members and revealed important clues on their origin and the gene duplications that generated them (Figure 4; Supplementary Figures S9–S10). More specifically, we observed several conserved syntenic blocks in jawed vertebrates, such as 1) *Plekha7*-*Sox6* and *Plekha6*-*Sox13*; 2) *Plekha7*-*Pik3c2a*, *Plekha6*-*Pik3c2b*, and *Plekha5*-*Pik3c2g*; 3) *Plekha7*-*Ppp1r15b*, *Plekha6*-*Ppp1r15b*, and *Plekha4*-*Ppp1r15a*; and 4) *Plekha7*-*Nucb2*-*Kcnj11* and *Plekha4*-*Nucb1*-*Kcnj14*. The *Plekha7*-*Pppr15b*, *Plekha7*-*Alx4*, and *Plekha6*-*Alx3* blocks are conserved only in Chondrichthyes and Actinopterygii, but subsequently lost in Coelacanths and Tetrapods (Figure 4; Supplementary Figures S9–S10). Overall, these results show that *Plekha7* exhibits the highest number of conserved syntenic blocks compared to its paralogs and that it is the only gene exhibiting conserved syntenic blocks with all the three members of the PLEKHA4/5/6/7 subfamily in jawed vertebrates.

Genomic neighborhood analyses revealed that the lamprey genes also displayed conserved synteny with jawed vertebrate *Plekha4,5,6,7* genes. Specifically, a *Petromyzon marinus gene* (XP_032804545) is in synteny with *Sox5*, similarly to *Sox6*-*Plekha7* and *Sox13*-*Plekha6*, and with *Kcna1*, similarly to *Plekha7*-*Kcnj11* and to *Plekha4*-*Kcnj14* in jawed vertebrates (Figure 4; Supplementary Figures S9–S10). A second *Petromyzon marinus* gene (XP_032815647) is in synteny with *Insc* and with *Slco1c1*, like the *Plekha7*-*Insc* and the *Plekha5*-*Slco1c1*, respectively, in teleosts (Figure 4; Supplementary Figures S9–S10). Lastly, the third *Petromyzon* gene we identified (LOC116956434; XP_032833925.1) does not exhibit any conserved synteny, either with the other two *Petromyzon* paralogs, or with any of the jawed vertebrate ones (Figure 4; Supplementary Figures S9–S10). These mixed syntenic relationships suggest that neither of these *Petromyzon* genes are clear *Plekha4,5,6,7* orthologs, further supporting the idea that these sequences are the result of lineage-specific duplications of a single gene, after the split of the jawed and jawless vertebrates. For this reason, we renamed these genes as *Plekha4/5/6/7a,b,c* (Figure 4 and throughout).

Analysis of the lancelet *Branchiostoma floridae Plekha4/5/6/7* gene revealed synteny with *Liprin-a*, which is conserved to the synteny observed between different *Plekha4/5/6/7* and *Liprin* genes in *Petromyzon* and in many teleosts (Figure 4; Supplementary Figures S9–S10). No syntenic relationships are conserved between non-chordate invertebrate *Plekha4/5/6/7* homologs and chordate genes of the subfamily (Supplementary Figure S9). Together, these findings indicate that the *Plekha4/5/6/7* genes originated independently in jawed and jawless vertebrates from a single ancestral gene that existed in the last common ancestor of all chordates.

### Regions of Significant Conservation Exist Within the PLEKHA4/5/6/7 Subfamily

**FIGURE 5:**
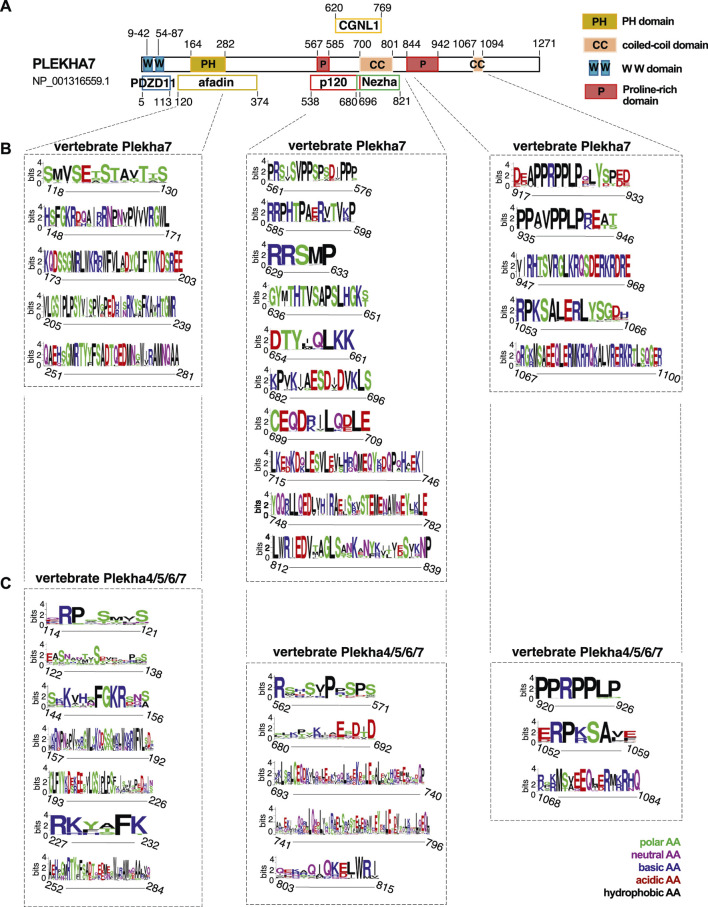
Vertebrate Plekha4/5/6/7 protein alignments reveal conserved domains. **(A)** Schematic representation of the domain structure of the human PLEKHA7, based on NCBI’s conserved domain database (CDD) and published work; key is shown on the top right. **(B,C)** Domain and sequence conservation of Plekha7 and Plekha4/5/6/7 proteins across vertebrates. Amino acid conservation was determined through WebLogo analysis of the respected vertebrate Plekha7 and Plekha4/5/6/7 multiple sequence alignments used for the phylogenetic analyses of Figure 2B and Supplementary Figure S2. The top conserved sequences are shown, as identified by the WebLogo bit scores. Conserved sequences are grouped based on their alignment to the PH/afadin, p120/Nezha/CGNL1, and C-terminal domains of the human PLEKHA7, as indicated by dotted lines and boxes. Numbers on the *x* axis under each sequence logo correspond to the amino acid positions of the human PLEKHA7 [see **(A)**]. Numbers in the *y* axis next to each sequence logo indicate the information content of a sequence position, in bits; by default, the height of the *y*-axis is the maximum entropy for protein sequences given by WebLogo (4.3 bits). Amino acid (AA) color code is shown on the bottom right.

The sequence conservation pattern between the members of the Plekha4/5/6/7 subfamily in vertebrates prompted us to investigate sequence similarities that might infer functionally important regions. The human PLEKHA7 is the best-studied member of the family, with well-identified domains that interact with cell-cell junction components, such as PDZD11, afadin, p120, Nezha, and CGNL1 (Figure 5A) (Meng et al., 2008; Pulimeno et al., 2011; Kurita et al., 2013; Guerrera et al., 2016; Sluysmans et al., 2021b). Amino acid conservation analysis revealed that the binding domains of PLEKHA7 with afadin, p120, Nezha, and CGNL1 are highly conserved in this protein throughout vertebrates (Figure 5B). However, comparisons of the Plekha4, Plekha5, and Plekha6 sequences aligned to p120, Nezha, and CGNL1 domains of Plekha7 yielded overall low amino acid conservation except for specific amino acid sites, which were conserved in all proteins (Figure 5C). Sequence conservation of the PH domain, which overlaps with the afadin-binding domain in PLEKHA7, was to some extent maintained across Plekha4/5/6/7 proteins, but to a far lesser extent across all Plekha proteins, further demonstrating the sequence divergence of the PLEKHA4/5/6/7 subfamily from the rest of the PLEKHA family (Figure 5C; Supplementary Figure S11A).

Notably, Plekha7 exhibits strong conservation in its C-terminus, specifically within its proline-rich and coiled-coiled domains (Figure 5B). Parts of these C-terminal proline-rich regions are also relatively well conserved across all Plekha4/5/6/7 members in vertebrates (Figure 5C). These regions are of unknown function in all Plekha4/5/6/7 members.

**FIGURE 6:**
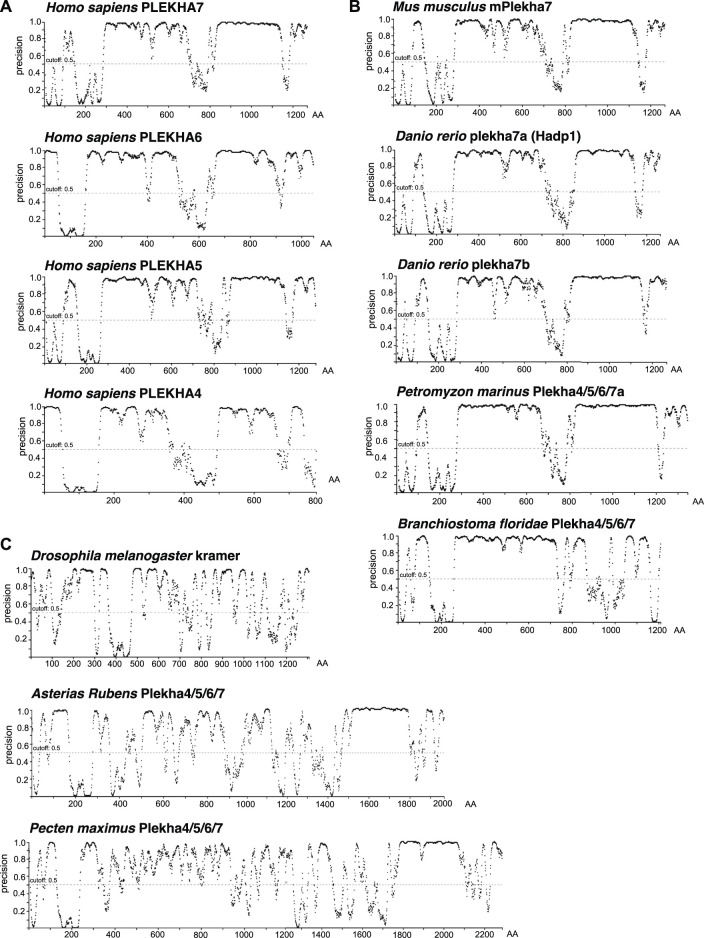
The chordate Plekha4/5/6/7 proteins possess structurally conserved intrinsically disordered regions (IDRs). Intrinsically disorder domain prediction analysis of **(A)** the human (*Homo sapiens*) PLEKHA4/5/6/7 proteins; **(B)** the mouse (*Mus musculus*) mPlekha7, zebrafish (*Danio rerio*) plekha7a and b, lamprey Plekha4/5/6/7a (*Petromyzon marinus* XP_032815647) and the lancelet Plekha4/5/6/7 (*Branchiostoma floridae* XP_035672282.1) proteins; and **(C)** the invertebrate *Pecten maximus* (XP_033751660.1) and *Asterias rubens* Plekha4/5/6/7 (XP_033645898.1) proteins retrieved in the BLAST searches (Figures 1B, 2A), as well as the *Drosophila melanogaster* kramer. Analysis was performed using the DISOPRED3 function of PSIPRED. Schematics representing the probability of a protein region being intrinsically disordered (“precision” on the *Y* axis). Numbers on the *x* axis denote amino acid positions (AA) of each protein.

Proline-rich domains are a typical characteristic of intrinsically disordered regions (IDRs) (Jarvelin et al., 2016). Recently, it has been reported that PLEKHA4 contains an IDR (Shami Shah et al., 2019). The significant conservation of the C-terminal regions, including the proline-rich regions, across Plekha4/5/6/7 members prompted us to investigate whether these regions are predicted to be IDRs. Disorder analysis revealed that all four Plekha4/5/6/7 contain two long IDRs, one between the PH domain and the coiled-coiled domain, and a second one after the coiled-coil domain, covering most of the C-terminal region (Figure 6A). The disorder predictions were almost identical in Plekha4/5/6/7 members from other vertebrates, such as mouse, zebrafish, or the jawless lamprey *Petromyzon marinus* (Figure 6B).

Disorder prediction analysis verified the presence of the first IDR in the *Branchiostoma floridae* Plekha4/5/6/7 sequence (Figure 6B). However, a sequence immediately adjacent to the C-terminus of the Plekha4/5/6/7 sequence is annotated as partial Plekha7-like sequence (XP_035672283.1; gene LOC118413180) (Supplementary Figure S11B). BLAST searches of this sequence revealed that it aligns considerably well to the vertebrate Plekha4/5/6/7 C-terminal sequences (Supplementary Table S10). Although these findings suggest that this partial sequence is the result of misannotation and could be part of the full-length lancelet Plekha4/5/6/7 protein, we could not reannotate the region to provide an open reading frame that includes both genes. Nevertheless, when we artificially merged the two protein sequences and performed the disorder prediction analysis, we were able to identify the missing C-terminus IDR of the lancelet XP_035672282.1 sequence (Supplementary Figure S11C). These data provide further, indirect support for the lancelet *Plekha4/5/6/7* gene being a representative of the last single common ancestor of the vertebrate *Plekha4,5,6,7* genes.

In contrast, disorder analysis of the few invertebrate sequences with homologies at their non-PH domain regions (Figure 1B **;** Supplementary Tables S6–S9), exhibited different patterns and low IDR predictions except for a region towards the extreme end of their C-terminus (Figure 6C). This region corresponds to the one found using BLAST searches (Figure 1A) and intronic analyses (Figure 3), suggesting that this may be a remnant of the ancestral invertebrate Plekha4/5/6/7 member. Still, disorder analysis of the *Drosophila* kramer produced entirely distinct patterns, compared to the chordate Plekha4/5/6/7 proteins, further underlining kramer’s sequence diversification (Figure 6C). Finally, the other members of the PLEKHA family (Plekha1, 2, 3, 8) showed lower disorder domain predictions, confirming their evolutionary and functional divergence from the PLEKHA4/5/6/7 subfamily (Supplementary Figure S11D).

**FIGURE 7:**
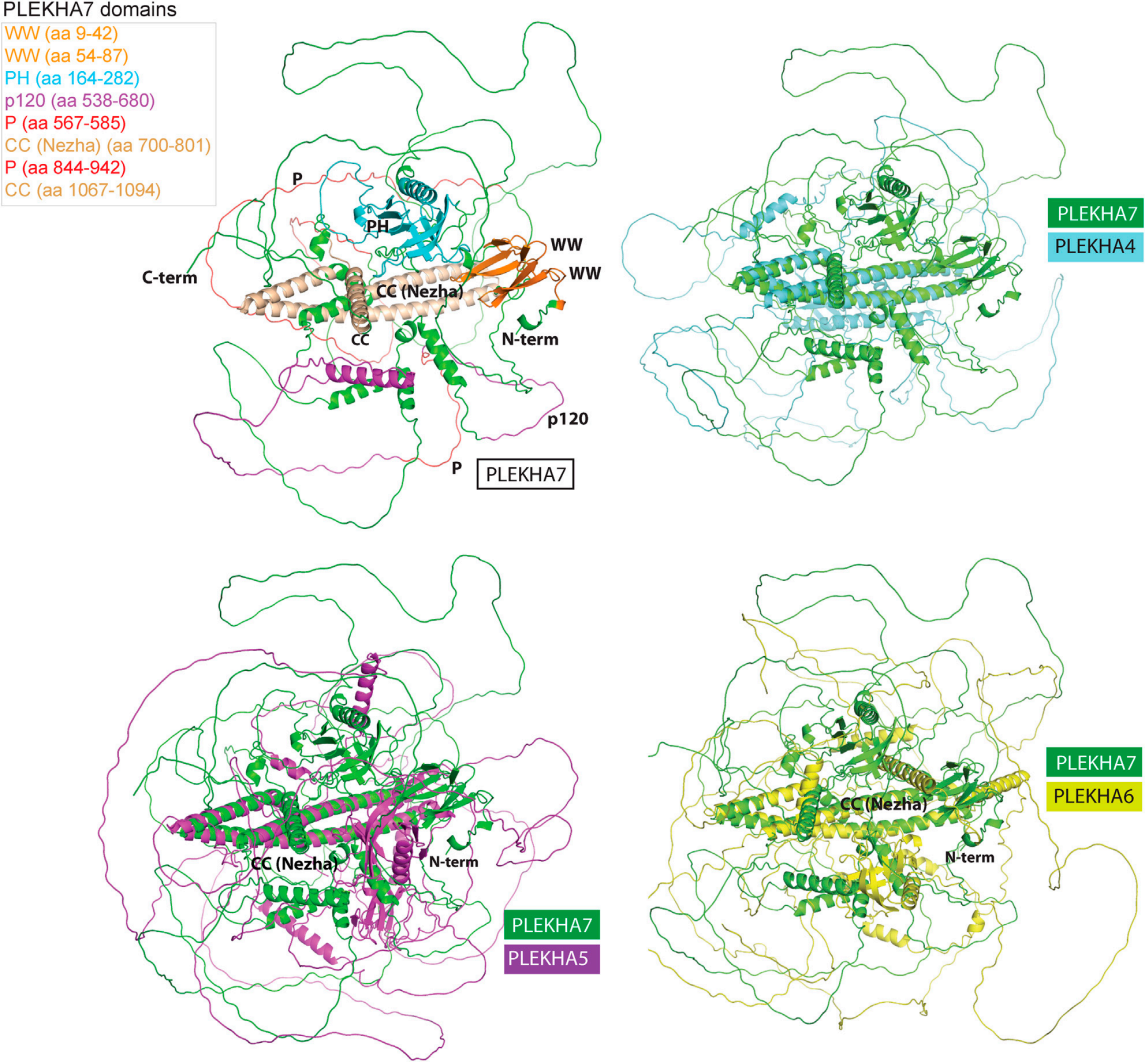
Structural superimpositions of human PLEKHA4,5,6,7 proteins reveal similarities of specific structural elements. Specific domains and functional regions of the PLEKHA7 protein molecule were mapped on to a theoretical three-dimensional model of the protein and color coded as shown in the left top panel. The PLEKHA7 model was superimposed to the PLEKHA4, PLEKHA5, and PLEKHA6 structural models to assess regional and domain similarities. The final figures were generated using Pymol ™ 1.7.4.5 (Delano Scientific) using the cartoon representations.

Computational predictions of the 3D structures of the human PLEKHA4/5/6/7 proteins confirm the presence of long IDR domains, which seem to engulf the structurally defined protein regions (Figure 7). Furthermore, structural superimposition of the different members reveals that in addition to the PH domain, the only other fully aligned region is the coiled-coiled domain in the C-terminal region of PLEKHA7 (Figure 7). This region is conserved in invertebrates (Figure 1A, Figure 3) and overlaps with the domain that: 1) PLEKHA7 binds to Nezha and the microtubules (Figure 5A) (Meng et al., 2008); 2) is critical for the apical junctional localization of PLEKHA7 and PLEKHA6 (Sluysmans et al., 2021a); and 3) mediates PLEKHA4 oligomerization (Shami Shah et al., 2019). Together, these results further support the idea that this region has been retained from the common invertebrate ancestor of the PLEKHA4/5/6/7 family, due to its functional significance.

## Discussion

**FIGURE 8:**
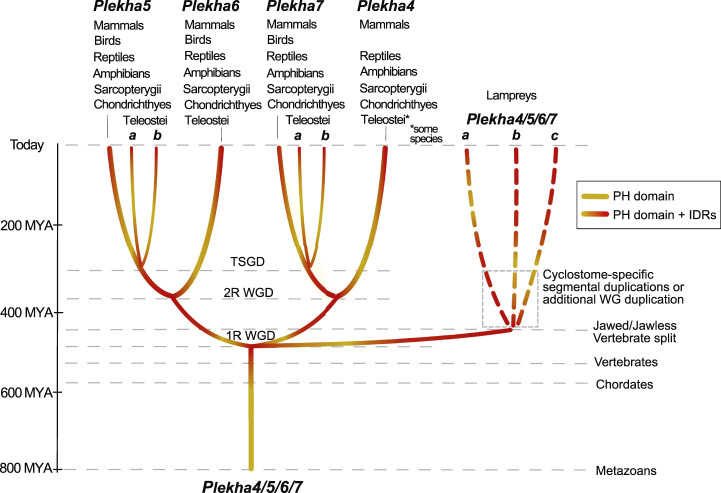
Hypothetical scenario depicting the evolutionary history of the Plekha4/5/6/7 protein subfamily in metazoans. A PEPP1-type PH domain acquired intrinsically disordered region (IDR) domains early in chordate evolution, in the last common ancestor of all vertebrates, and subsequently underwent gene duplications. In jawed vertebrates, a first gene duplication gave rise to the *Plekha4/7* and *Plekha5/6* ancestral genes and a second duplication to the four modern *Plekha4,5,6,7* genes. These duplications are consistent with the vertebrate 1R and 2R whole genome duplications (WGD) hypothesis. An additional gene duplication consistent with the Teleost-specific whole-genome duplication (TSGD), resulted in *Plekha7* and *Plekha5* having two copies in these species (a and b). In parallel, gene duplications that occurred independently in jawless vertebrates, potentially due to cyclostome-specific segmental duplications or additional whole-genome (WG) duplication, gave rise to three genes in lampreys that we name *Plekha4/5/6/7a,b,c*. Approximate timeline of events is shown on the left and with parallel dotted lines, indicating major events throughout metazoan evolution. Taxa names atop each of the four *Plekha4,5,6,7* genes refer to their presence in extant jawed vertebrate species from those taxa.

A multiplicity of studies has shown that Plekha7 is an AJ component critical both for the integrity of the complex, as well as for a wide range of cellular functions in mammalian cells. Our analyses show that the *Plekha7*, *Plekha4*, *Plekha5*, and *Plekha6* genes are jawed vertebrate-specific paralogs. This statement is supported by multiple pieces of evidence that identified: 1) a single gene in cephalochordate and tunicate species, which we rename as *Plekha4/5/6/7*; and 2) three genes in the jawless vertebrate lampreys, which based on their mixed phylogenetic and syntenic patterns are lineage-specific, and thus we rename as *Plekha4/5/6/7a,b,c* (Figures 2A, 3, 4, 6, 8). These observations support a scenario that describes the evolution of the *Plekha4,5,6,7* genes. According to this scenario, a single gene was present in the ancestor of all chordates. That gene remained single in cephalochordates and tunicates; however, it underwent two rounds of duplication in jawed vertebrates, after their split from invertebrates, the first resulting in the *Plekha4/7* and *Plekha5/6* gene clades, and the second resulting in the four modern *Plekha4*, *Plekha5*, *Plekha6*, and *Plekha7* jawed vertebrate–specific genes (Figure 8). The ancestral single chordate gene triplicated independently in jawless vertebrates, after their split from the jawed vertebrate lineage, resulting in the jawless vertebrate–specific *Plekha4/5/6/7a,b,c* genes (Figure 8).

These gene duplication events are consistent with the proposed two rounds (1R, 2R) of whole genome-duplication (WGD) events that occurred early in vertebrate evolution (Abi-Rached et al., 2002; Dehal and Boore, 2005; Nakatani et al., 2007; Putnam et al., 2008; Lagman et al., 2013; Marletaz et al., 2018; Ocampo Daza and Larhammar, 2018; Sacerdot et al., 2018; Simakov et al., 2020; Nakatani et al., 2021), as well as with previous studies proposing that lamprey genomes independently underwent polyploidizations, by either a WGD (Mehta et al., 2013; Coppola and Waxman, 2021), hexaploidization *via* hybridization between tetraploid and diploid lineages (Nakatani et al., 2021), or extensive segmental duplications (Smith and Keinath, 2015). Our data cannot formally deduce which particular scenario explains the evolution of the lamprey *Plekha4/5/6/7a,b,c* genes, because all alternatives can accommodate our observations.

Furthermore, in ray-finned fishes, there was an additional duplication that produced two *plekha7* and *plekha5* copies (*a* and *b*). This finding reflects the Teleostei-specific whole-genome duplication event (TSGD) (Taylor et al., 2003; Christoffels et al., 2004; Jaillon et al., 2004; Glasauer and Neuhauss, 2014) (Figure 8). Considering that the teleost *plekha7b* copy retains alternatively spliced isoforms like the mammalian genes, whereas *plekha7a* does not (Supplementary Table S1B), and that *plekha7a* copies have longer branches in all phylogenetic trees (Figure 2 and Supplementary Figure S4), we infer that the latter genes diversified in teleosts, whereas the *plekha7b* ones retain ancestral functional roles. In contrast, duplicate copies of *plekha4* and *plekha6* were not retained in teleosts and *plekha5* copies were retained only in some of those species, suggesting different evolutionary pressures acting on those genes. Furthermore, the river trout has additional copies only of *plekha7* (Supplementary Figure S4), probably due to the salmonid-specific whole-genome duplication (SSGD) (Lien et al., 2016). These observations insinuate that Plekha7 may have further functional roles allowing evolution of paralogous copies, as opposed to the other members of the subfamily, similarly to findings in other teleost gene families (Guo, 2017).

The latter supposition of differential evolutionary forces agrees with the observation that *Plekha7* is the most conserved member of the subfamily, retaining several sequence and genomic features of the ancestral *Plekha4/5/6/7* gene. In contrast *Plekha7*’s closest paralog, *Plekha4*, is the least conserved vertebrate member of the subfamily, as demonstrated by its high sequence divergence and gene loss in several taxonomic groups (Figures 1A,B), which may imply functional diversification.

Considering that all identified chordate proteins contain a PH domain and a highly conserved coiled-coil domain flanked by two extensive IDRs, we suggest that the common ancestor of chordates had a gene with similar domain organization. The question that arises from this supposition is when and how did this domain combination arise during evolution. Based on our data, the PEPP-type PH domain, characteristic of the Plekha4,5,6,7 proteins, exists in almost all extant animal lineages (Figures 1A,B). Furthermore, this type of PH domain together with a coiled-coil region are present on a single molecule in a few divergent non-chordate species (Figures 1A,B). In the latter species, these proteins also contain a portion of the extreme C-terminal domain shared by all Plekha4, 5, 6, 7 proteins (Figure 1A). Although independent domain acquisition cannot be formally excluded, a more plausible explanation of these observations is that these domains/regions were fused together once during animal evolution and were subsequently lost in most invertebrates.

Although the PH-coiled-coil domain combination is present in all vertebrates and in the few invertebrates discussed above, the presence of the two extensive IDRs found in all Plekha4,5,6,7 members is more enigmatic. The two IDRs, flanking the coiled-coil domain, are highly divergent in sequence even within the vertebrate genes and this level of divergence may obscure any identifiable phylogenetic signal in non-chordate animals. If this explanation holds true, then it is conceivable to speculate that one or both IDRs were part of the ancestral molecule that gave rise to the Plekha4,5,6,7 members. During the evolution of these molecules, the IDRs might have been less constraint by functional requirements compared to other regions and were copiously diversified. Alternatively, but perhaps not mutually exclusive, the IDRs might have appeared in the common single ancestral chordate gene “*de novo*” using a mechanism like the one proposed by (Carvunis et al., 2012). The finding of altered intron phases throughout the evolution of the *Plekha4/5/6/7* genes and their extreme alternative splicing (Supplementary Table S1B) reflect a highly dynamic structure, which could result in different evolutionary pressures, alternative gene structures, and gain or loss of different DNA fragments. Although these speculations rationalize the emergence of the Plekha4/5/6/7 IDRs in vertebrates, the current sequencing and genomic data are not sufficient to fully support either of these ideas, since this information may have existed in the last common ancestor of vertebrates and is lost in the extant genomes. Nevertheless, the acquisition of these extensive IDRs by the vertebrate Plekha4/5/6/7 proteins seems to be a key event in their evolution. Indeed, these IDRs are topologically and structurally conserved among all four members of the subfamily and across different vertebrate species analyzed, denoting their potential functional significance, which warrants further exploration.

Our findings on the evolution and multiplication of the *Plekha4,5,6,7* genes in vertebrates agree with the expanded compositional and functional repertoire of the adherens junctions and all their functional components in vertebrates. However, the distinctive patterns of domain and sequence conservation of the Plekha4,5,6,7 proteins in jawed vertebrates (Figures 5–7), as well as recently published work (Shami Shah et al., 2019; Sluysmans et al., 2021a), also suggest functional diversification, a concept that warrants experimental validation. Considering the extend of sequence diversification and gene birth-and-death rate especially in invertebrates, it is rational to wonder whether the functions currently attributed to the Plekha4,5,6,7 proteins are also vertebrate-specific. For example, we have shown that PLEKHA7 defines an apical p120 junctional complex that suppresses pro-growth behavior of mammalian epithelial cells (Kourtidis et al., 2015). p120 is one of the four vertebrate members of the delta catenin family that is essential for survival in vertebrates, whereas the single delta catenin member is dispensable in invertebrates (Myster et al., 2003; Ciesiolka et al., 2004; Fang et al., 2004). Based on the above, it is tempting to examine whether Plekha7 evolved as a rheostat of these pro-survival functions of p120 in vertebrates, to maintain cellular homeostasis. Along the same lines, we have shown that PLEKHA7 confers this homeostatic function through recruitment of the RNAi machinery to mammalian epithelial AJs (Kourtidis et al., 2015; Kourtidis et al., 2017a; Nair-Menon et al., 2020). It would be interesting to examine whether this RNAi localization to the AJs is also vertebrate-specific. These are concepts important to elucidate, since they would also determine whether invertebrate models, such as *Drosophila*, are appropriate to study functions, like localization of RNAi to the junctions, or whether vertebrate-specific models would be required, instead.

The complex evolutionary history of the *Plekha4,5,6,7* genes contrasts with the other members of the PLEKHA family, which apparently followed a very different evolutionary trajectory. The PLEKHA1/2 and PLEKHA3/8 subfamilies were products of evolutionary innovation (duplication of a single PH domain and fusion with a Glycolipid transfer protein domain, respectively; Figure 1A), which occurred early in animal evolution and remained largely unchanged, after duplication of the genes in vertebrates. However, the differences between the subfamilies are not restricted to their evolutionary paths but expand to their only common region, the PH domains. Apparently, there are three types of PH domains within the PLEKHA family (TAPP, FAPP, and PEPP; Figure 1A), which do not seem to share a most recent common evolutionary ancestor. Although the origin and evolution of the PH domains are beyond the scope of the current work, collectively our results demonstrate that the Plekha proteins belong to three separate subfamilies, PLEKHA4/5/6/7, PLEKHA1/2, and PLEKHA3/8, which given their differences might need to be considered as separate families. In summary, our work unravels the intriguing evolutionary history of Plekha7 and of the Plekha proteins and puts forth information that may further stimulate functional studies of this relatively unexplored group of proteins.

## Data Availability

The datasets presented in this study can be found in online repositories. The names of the repository/repositories and accession number(s) can be found in the article/Supplementary Material.
