# Supplementary material for: Origin and Evolution of the Multifaceted Adherens Junction Component Plekha7
Source: Front Cell Dev Biol. 2022 Mar 23;10:856975. doi: 10.3389/fcell.2022.856975 (PMC8983885; doi:10.3389/fcell.2022.856975)
Supplement: Supplementary file 5 [file DataSheet1.pdf]

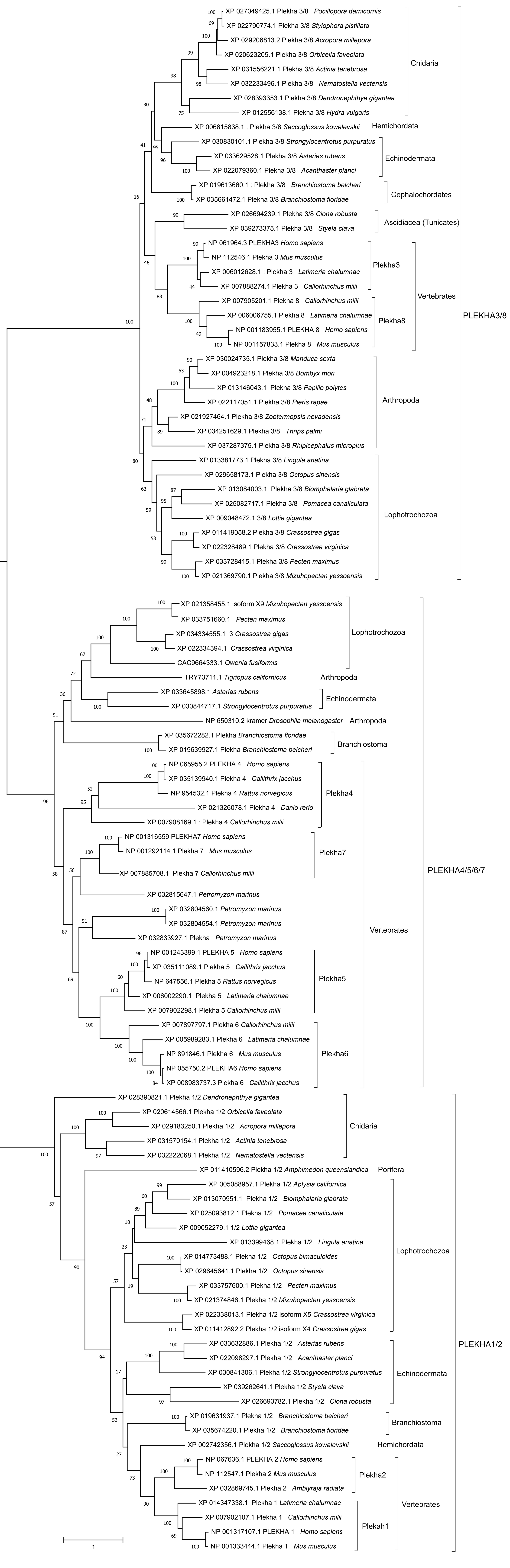
XP 027049425.1 Plekha 3/8 Pocillopora damicornis
XP 022790774.1 Plekha 3/8 Stylophora pistillata
XP 029206813.2 Plekha 3/8 Acropora millepora
XP 020623205.1 Plekha 3/8 Orbicella faveolata
XP 031556221.1 Plekha 3/8 Actinia tenebrosa
XP 032233496.1 Plekha 3/8 Nematostella vectensis
XP 028393353.1 Plekha 3/8 Dendronephthya gigantea
XP 012556138.1 Plekha 3/8 Hydra vulgaris
Cnidaria
XP 006815838.1 : Plekha 3/8 Saccoglossus kowalevskii
Hemichordata
XP 030830101.1 Plekha 3/8 Strongylocentrotus purpuratus
XP 033629528.1 Plekha 3/8 Asterias rubens
XP 022079360.1 Plekha 3/8 Acanthaster planci
Echinodermata
XP 019613660.1 : Plekha 3/8 Branchiostoma belcheri
XP 035661472.1 Plekha 3/8 Branchiostoma floridae
Cephalochordates
XP 026694239.1 Plekha 3/8 Ciona robusta
XP 039273375.1 Plekha 3/8 Styela clava
Ascidiacea (Tunicates)
NP 061964.3 PLEKHA3 Homo sapiens
NP 112546.1 Plekha 3 Mus musculus
XP 006012628.1 : Plekha 3 Latimeria chalumnae
XP 007888274.1 Plekha 3 Callorhinchus milii
Plekha3
XP 007905201.1 Plekha 8 Callorhinchus milii
XP 006006755.1 Plekha 8 Latimeria chalumnae
NP 001183955.1 PLEKHA 8 Homo sapiens
NP 001157833.1 Plekha 8 Mus musculus
Plekha8
Vertebrates
PLEKHA3/8
XP 030024735.1 Plekha 3/8 Manduca sexta
XP 004923218.1 Plekha 3/8 Bombyx mori
XP 013146043.1 Plekha 3/8 Papilio polytes
XP 022117051.1 Plekha 3/8 Pieris rapae
XP 021927464.1 Plekha 3/8 Zootermopsis nevadensis
XP 034251629.1 Plekha 3/8 Thrips palmi
XP 037287375.1 Plekha 3/8 Rhipicephalus microplus
Arthropoda
XP 013381773.1 Plekha 3/8 Lingula anatina
XP 029658173.1 Plekha 3/8 Octopus sinensis
XP 013084003.1 Plekha 3/8 Biomphalaria glabrata
XP 025082717.1 Plekha 3/8 Pomacea canaliculata
XP 009048472.1 3/8 Lottia gigantea
XP 011419058.2 Plekha 3/8 Crassostrea gigas
XP 022328489.1 Plekha 3/8 Crassostrea virginica
XP 033728415.1 Plekha 3/8 Pecten maximus
XP 021369790.1 Plekha 3/8 Mizuhopecten yessoensis
Lophotrochozoa
XP 021358455.1 isoform X9 Mizuhopecten yessoensis
XP 033751660.1 Pecten maximus
XP 034334555.1 3 Crassostrea gigas
XP 022334394.1 Crassostrea virginica
CAC9664333.1 Owenia fusiformis
Lophotrochozoa
TRY73711.1 Tigriopus californicus
Arthropoda
XP 033645898.1 Asterias rubens
XP 030844717.1 Strongylocentrotus purpuratus
Echinodermata
NP 650310.2 kramer Drosophila melanogaster
Arthropoda
XP 035672282.1 Plekha Branchiostoma floridae
XP 019639927.1 Plekha Branchiostoma belcheri
Branchiostoma
NP 065955.2 PLEKHA 4 Homo sapiens
XP 035139940.1 Plekha 4 Callithrix jacchus
NP 954532.1 Plekha 4 Rattus norvegicus
XP 021326078.1 Plekha 4 Danio rerio
XP 007908169.1 : Plekha 4 Callorhinchus milii
Plekha4
NP 001316559 PLEKHA7 Homo sapiens
NP 001292114.1 Plekha 7 Mus musculus
XP 007885708.1 Plekha 7 Callorhinchus milii
Plekha7
XP 032815647.1 Petromyzon marinus
XP 032804560.1 Petromyzon marinus
XP 032804554.1 Petromyzon marinus
XP 032833927.1 Plekha Petromyzon marinus
NP 001243399.1 PLEKHA 5 Homo sapiens
XP 035111089.1 Plekha 5 Callithrix jacchus
NP 647556.1 Plekha 5 Rattus norvegicus
XP 006002290.1 Plekha 5 Latimeria chalumnae
XP 007902298.1 Plekha 5 Callorhinchus milii
Plekha5
XP 007897797.1 Plekha 6 Callorhinchus milii
XP 005989283.1 Plekha 6 Latimeria chalumnae
NP 891846.1 Plekha 6 Mus musculus
NP 055750.2 PLEKHA6 Homo sapiens
XP 008983737.3 Plekha 6 Callithrix jacchus
Plekha6
Vertebrates
PLEKHA4/5/6/7
XP 028390821.1 Plekha 1/2 Dendronephthya gigantea
XP 020614566.1 Plekha 1/2 Orbicella faveolata
XP 029183250.1 Plekha 1/2 Acropora millepora
XP 031570154.1 Plekha 1/2 Actinia tenebrosa
XP 032222068.1 Plekha 1/2 Nematostella vectensis
Cnidaria
XP 011410596.2 Plekha 1/2 Amphimedon queenslandica
Porifera
XP 005088957.1 Plekha 1/2 Aplysia californica
XP 013070951.1 Plekha 1/2 Biomphalaria glabrata
XP 025093812.1 Plekha 1/2 Pomacea canaliculata
XP 009052279.1 1/2 Lottia gigantea
XP 013399468.1 Plekha 1/2 Lingula anatina
XP 014773488.1 Plekha 1/2 Octopus bimaculoides
XP 029645641.1 Plekha 1/2 Octopus sinensis
XP 033757600.1 Plekha 1/2 Pecten maximus
XP 021374846.1 Plekha 1/2 Mizuhopecten yessoensis
XP 022338013.1 Plekha 1/2 isoform X5 Crassostrea virginica
XP 011412892.2 Plekha 1/2 isoform X4 Crassostrea gigas
Lophotrochozoa
XP 033632886.1 Plekha 1/2 Asterias rubens
XP 022098297.1 Plekha 1/2 Acanthaster planci
XP 030841306.1 Plekha 1/2 Strongylocentrotus purpuratus
Echinodermata
XP 039262641.1 Plekha 1/2 Styela clava
XP 026693782.1 Plekha 1/2 Ciona robusta
XP 019631937.1 Plekha 1/2 Branchiostoma belcheri
XP 035674220.1 Plekha 1/2 Branchiostoma floridae
Branchiostoma
XP 002742356.1 Plekha 1/2 Saccoglossus kowalevskii
Hemichordata
NP 067636.1 PLEKHA 2 Homo sapiens
NP 112547.1 Plekha 2 Mus musculus
XP 032869745.1 Plekha 2 Amblyraja radiata
Plekha2
XP 014347338.1 Plekha 1 Latimeria chalumnae
XP 007902107.1 Plekha 1 Callorhinchus milii
NP 001317107.1 PLEKHA 1 Homo sapiens
NP 001333444.1 Plekha 1 Mus musculus
Plekah1
Vertebrates
PLEKHA1/2
1

Supplementary Figure S1. Uncompressed phylogenetic tree corresponding to **Figure 2A**, showing the evolutionary relationships of all the members of the PLEKHA protein family. The tree was inferred by the Maximum Likelihood methods using JTT matrix-based model. The tree with the highest log likelihood (-123550.77) is shown. Bootstrap values indicate the percentage of 1000 replicates in which the associated taxa clustered in the resulting tree. A discrete Gamma distribution was used to model evolutionary rate differences among sites (5 categories (+*G*, parameter = 2.0629)). The tree is drawn to scale, with branch lengths measured in the number of substitutions per site. This analysis involved 107 amino acid sequences. All positions containing gaps and missing data were eliminated (complete deletion option) and a total of 77 positions were used.

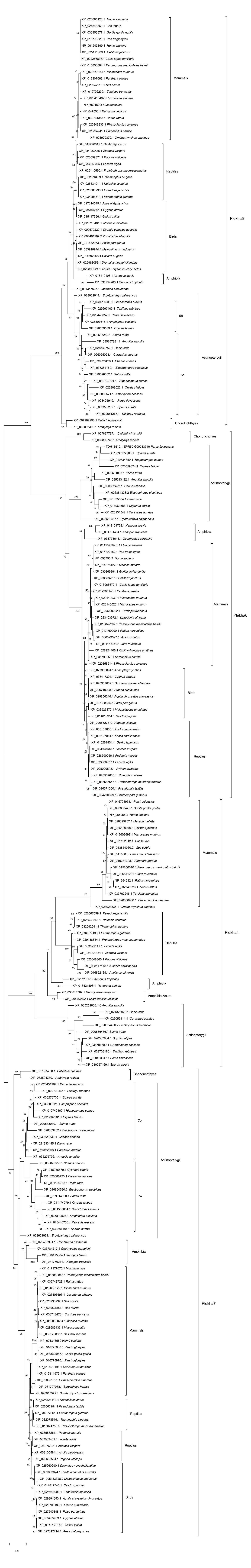

Mammals
Reptiles
Birds
Amphibia
5b
5a
Actinopterygii
Chondrichthyes
Plekha5
Chondrichthyes
Actinopterygii
Amphibia
Mammals
Birds
Reptiles
Plekha6
Mammals
Reptiles
Plekha4
Amphibia
Amphibia-Anura
Actinopterygii
Chondrichthyes
7b
7a
Actinopterygii
Amphibia
Plekha7
Mammals
Reptiles
Reptiles
Birds
0.20

Supplementary Figure S2. Evolutionary relationships of the vertebrate Plekha4,5,6,7 proteins. This is the expanded form of the tree shown in **Figure 2B**, in which the major clades were compressed for better visualization. The phylogenetic relationships between the Plekha4,5,6,7 proteins were inferred using the Maximum Likelihood methods based on the JTT matrix-based model, with complete deletion of amino acid positions containing gaps. There was a total of 245 positions in the final dataset. The tree with the highest log likelihood (-16497.96) is shown. Bootstrap values (numbers at nodes) indicate the percentage of 1000 replicates in which the associated taxa clustered in the resulting tree. A discrete Gamma distribution was used to model evolutionary rate differences among sites (5 categories (+*G*, parameter = 1.5563)). The tree is drawn to scale, with branch lengths measured in the number of substitutions per site. This analysis involved 231 amino acid sequences. Teleost-specific copies are indicated by a and b.

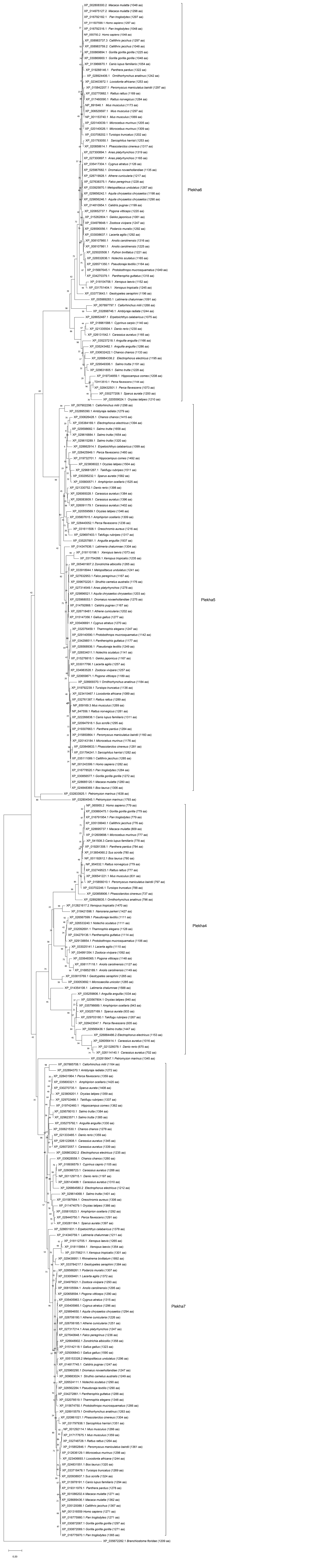
Plekha6
Plekha5
Plekha4
Plekha7
0.20

Supplementary Figure S3. Evolutionary relationships of the Plekha4,5,6,7 proteins, including the *Branchiostoma floridae* sequence as outgroup. The evolutionary history was inferred using the Neighbor-Joining method. The optimal tree is shown. The percentage of replicate trees in which the associated taxa clustered together in the bootstrap test (1000 replicates) are shown next to the branches. The tree is drawn to scale, with branch lengths in the same units as those of the evolutionary distances used to infer the phylogenetic tree. The evolutionary distances were computed using the JTT matrix-based method and are in the units of the number of amino acid substitutions per site. The rate variation among sites was modeled with a gamma distribution (shape parameter = 0.99). This analysis involved 262 amino acid sequences. All positions containing gaps and missing data were eliminated (complete deletion option). There was a total of 219 positions in the final dataset.

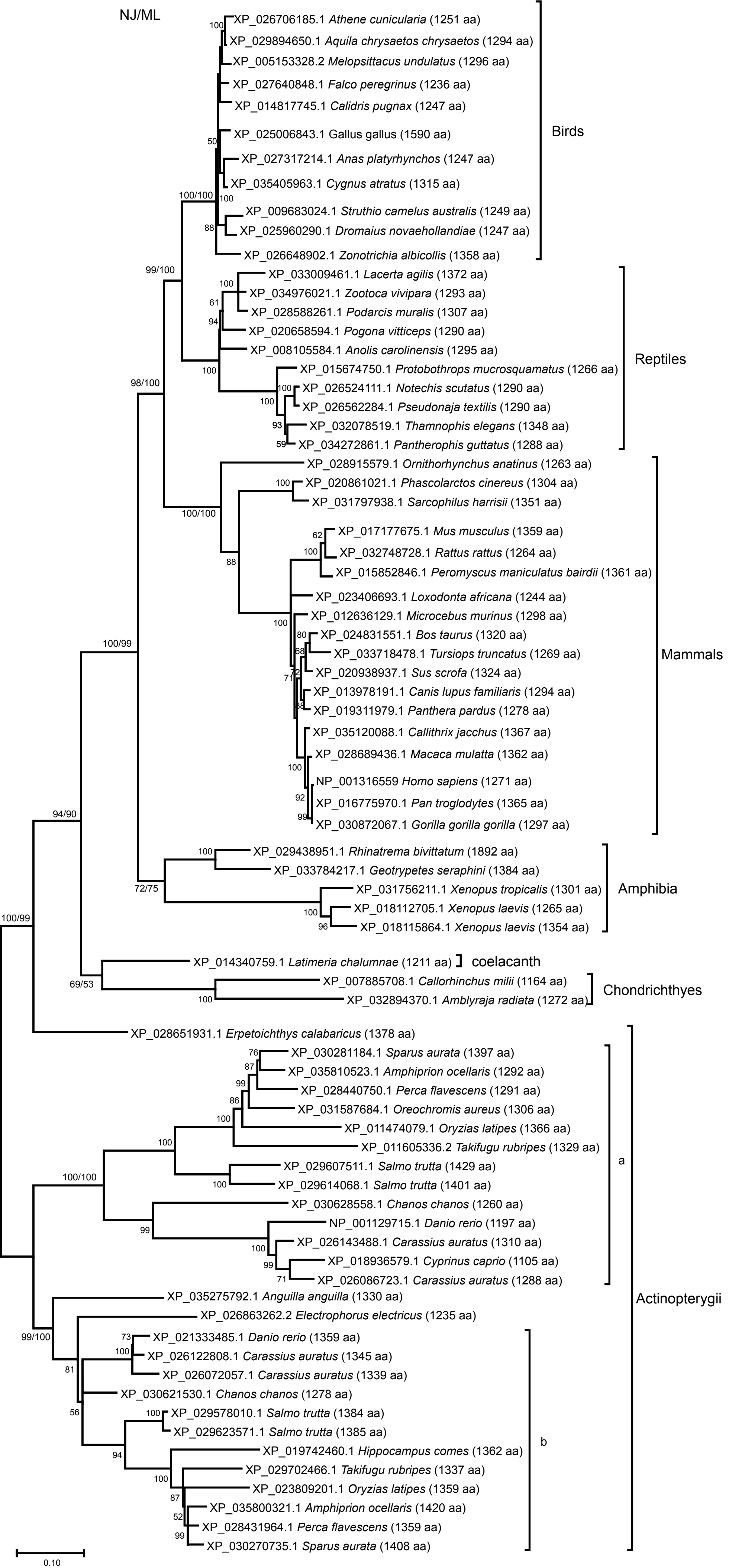
NJ/ML
XP_026706185.1 Athene cunicularia (1251 aa)
XP_029894650.1 Aquila chrysaetos chrysaetos (1294 aa)
XP_005153328.2 Melopsittacus undulatus (1296 aa)
XP_027640848.1 Falco peregrinus (1236 aa)
XP_014817745.1 Calidris pugnax (1247 aa)
XP_025006843.1 Gallus gallus (1590 aa)
XP_027317214.1 Anas platyrhynchos (1247 aa)
XP_035405963.1 Cygnus atratus (1315 aa)
XP_009683024.1 Struthio camelus australis (1249 aa)
XP_025960290.1 Dromaius novaehollandiae (1247 aa)
XP_026648902.1 Zonotrichia albicollis (1358 aa)
Birds
XP_033009461.1 Lacerta agilis (1372 aa)
XP_034976021.1 Zootoca vivipara (1293 aa)
XP_028588261.1 Podarcis muralis (1307 aa)
XP_020658594.1 Pogona vitticeps (1290 aa)
XP_008105584.1 Anolis carolinensis (1295 aa)
XP_015674750.1 Protobothrops mucrosquamatus (1266 aa)
XP_026524111.1 Notechis scutatus (1290 aa)
XP_026562284.1 Pseudonaja textilis (1290 aa)
XP_032078519.1 Thamnophis elegans (1348 aa)
XP_034272861.1 Pantherophis guttatus (1288 aa)
Reptiles
XP_028915579.1 Ornithorhynchus anatinus (1263 aa)
XP_020861021.1 Phascolarctos cinereus (1304 aa)
XP_031797938.1 Sarcophilus harrisii (1351 aa)
XP_017177675.1 Mus musculus (1359 aa)
XP_032748728.1 Rattus rattus (1264 aa)
XP_015852846.1 Peromyscus maniculatus bairdii (1361 aa)
XP_023406693.1 Loxodonta africana (1244 aa)
XP_012636129.1 Microcebus murinus (1298 aa)
XP_024831551.1 Bos taurus (1320 aa)
XP_033718478.1 Tursiops truncatus (1269 aa)
XP_020938937.1 Sus scrofa (1324 aa)
XP_013978191.1 Canis lupus familiaris (1294 aa)
XP_019311979.1 Panthera pardus (1278 aa)
XP_035120088.1 Callithrix jacchus (1367 aa)
XP_028689436.1 Macaca mulatta (1362 aa)
NP_001316559 Homo sapiens (1271 aa)
XP_016775970.1 Pan troglodytes (1365 aa)
XP_030872067.1 Gorilla gorilla gorilla (1297 aa)
Mammals
XP_029438951.1 Rhinatrema bivittatum (1892 aa)
XP_033784217.1 Geotrypetes seraphini (1384 aa)
XP_031756211.1 Xenopus tropicalis (1301 aa)
XP_018112705.1 Xenopus laevis (1265 aa)
XP_018115864.1 Xenopus laevis (1354 aa)
Amphibia
XP_014340759.1 Latimeria chalumnae (1211 aa)
coelacanth
XP_007885708.1 Callorhinchus milii (1164 aa)
XP_032894370.1 Amblyraja radiata (1272 aa)
Chondrichthyes
XP_028651931.1 Erpetoichthys calabaricus (1378 aa)
XP_030281184.1 Sparus aurata (1397 aa)
XP_035810523.1 Amphiprion ocellaris (1292 aa)
XP_028440750.1 Perca flavescens (1291 aa)
XP_031587684.1 Oreochromis aureus (1306 aa)
XP_011474079.1 Oryzias latipes (1366 aa)
XP_011605336.2 Takifugu rubripes (1329 aa)
XP_029607511.1 Salmo trutta (1429 aa)
XP_029614068.1 Salmo trutta (1401 aa)
XP_030628558.1 Chanos chanos (1260 aa)
NP_001129715.1 Danio rerio (1197 aa)
XP_026143488.1 Carassius auratus (1310 aa)
XP_018936579.1 Cyprinus caprio (1105 aa)
XP_026086723.1 Carassius auratus (1288 aa)
a
XP_035275792.1 Anguilla anguilla (1330 aa)
XP_026863262.2 Electrophorus electricus (1235 aa)
XP_021333485.1 Danio rerio (1359 aa)
XP_026122808.1 Carassius auratus (1345 aa)
XP_026072057.1 Carassius auratus (1339 aa)
XP_030621530.1 Chanos chanos (1278 aa)
XP_029578010.1 Salmo trutta (1384 aa)
XP_029623571.1 Salmo trutta (1385 aa)
XP_019742460.1 Hippocampus comes (1362 aa)
XP_029702466.1 Takifugu rubripes (1337 aa)
XP_023809201.1 Oryzias latipes (1359 aa)
XP_035800321.1 Amphiprion ocellaris (1420 aa)
XP_028431964.1 Perca flavescens (1359 aa)
XP_030270735.1 Sparus aurata (1408 aa)
b
Actinopterygii
0.10

Supplementary Figure S4. Phylogenetic reconstruction of Plekha7 proteins reveals orthology within vertebrates. Phylogenetic relationships between the homologs were determined using the Neighbor-Joining method and the Maximum Likelihood methods based on the JTT matrix-based model, with complete deletion of amino acid positions containing gaps. There was a total of 718 positions in the final dataset. The optimal tree and the tree with the highest log likelihood (-21232.73) are shown. Bootstrap values (numbers at nodes for NJ and ML) indicate the percentage of 1000 replicates in which the associated taxa clustered in the resulting tree. A discrete Gamma distribution was used to model evolutionary rate differences among sites (5 categories (+G, parameter = 0.9165)). The rate variation model allowed for some sites to be evolutionarily invariable ([+I], 12.88% sites). The trees are drawn to scale, with branch lengths measured in the number of substitutions per site. Teleost-specific copies are indicated by a and b.

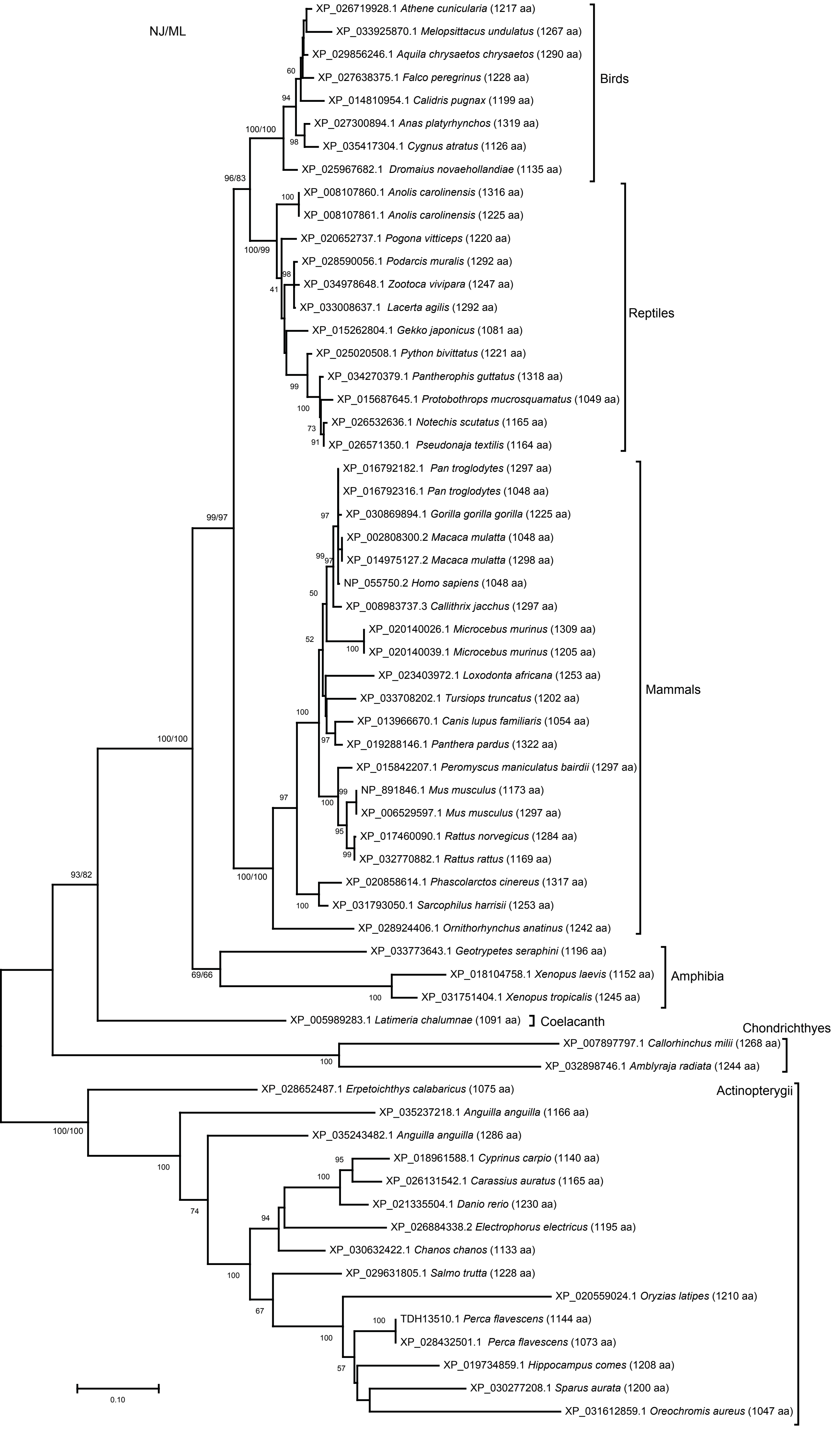
NJ/ML
XP_026719928.1 Athene cunicularia (1217 aa)
XP_033925870.1 Melopsittacus undulatus (1267 aa)
XP_029856246.1 Aquila chrysaetos chrysaetos (1290 aa)
XP_027638375.1 Falco peregrinus (1228 aa)
XP_014810954.1 Calidris pugnax (1199 aa)
XP_027300894.1 Anas platyrhynchos (1319 aa)
XP_035417304.1 Cygnus atratus (1126 aa)
XP_025967682.1 Dromaius novaehollandiae (1135 aa)
Birds
XP_008107860.1 Anolis carolinensis (1316 aa)
XP_008107861.1 Anolis carolinensis (1225 aa)
XP_020652737.1 Pogona vitticeps (1220 aa)
XP_028590056.1 Podarcis muralis (1292 aa)
XP_034978648.1 Zootoca vivipara (1247 aa)
XP_033008637.1 Lacerta agilis (1292 aa)
XP_015262804.1 Gekko japonicus (1081 aa)
XP_025020508.1 Python bivittatus (1221 aa)
XP_034270379.1 Pantherophis guttatus (1318 aa)
XP_015687645.1 Protobothrops mucrosquamatus (1049 aa)
XP_026532636.1 Notechis scutatus (1165 aa)
XP_026571350.1 Pseudonaja textilis (1164 aa)
Reptiles
XP_016792182.1 Pan troglodytes (1297 aa)
XP_016792316.1 Pan troglodytes (1048 aa)
XP_030869894.1 Gorilla gorilla gorilla (1225 aa)
XP_002808300.2 Macaca mulatta (1048 aa)
XP_014975127.2 Macaca mulatta (1298 aa)
NP_055750.2 Homo sapiens (1048 aa)
XP_008983737.3 Callithrix jacchus (1297 aa)
XP_020140026.1 Microcebus murinus (1309 aa)
XP_020140039.1 Microcebus murinus (1205 aa)
XP_023403972.1 Loxodonta africana (1253 aa)
XP_033708202.1 Tursiops truncatus (1202 aa)
XP_013966670.1 Canis lupus familiaris (1054 aa)
XP_019288146.1 Panthera pardus (1322 aa)
XP_015842207.1 Peromyscus maniculatus bairdii (1297 aa)
NP_891846.1 Mus musculus (1173 aa)
XP_006529597.1 Mus musculus (1297 aa)
XP_017460090.1 Rattus norvegicus (1284 aa)
XP_032770882.1 Rattus rattus (1169 aa)
XP_020858614.1 Phascolarctos cinereus (1317 aa)
XP_031793050.1 Sarcophilus harrisii (1253 aa)
XP_028924406.1 Ornithorhynchus anatinus (1242 aa)
Mammals
XP_033773643.1 Geotrypetes seraphini (1196 aa)
XP_018104758.1 Xenopus laevis (1152 aa)
XP_031751404.1 Xenopus tropicalis (1245 aa)
Amphibia
XP_005989283.1 Latimeria chalumnae (1091 aa)
Coelacanth
Chondrichthyes
XP_007897797.1 Callorhinchus milii (1268 aa)
XP_032898746.1 Amblyraja radiata (1244 aa)
XP_028652487.1 Erpetoichthys calabaricus (1075 aa)
Actinopterygii
XP_035237218.1 Anguilla anguilla (1166 aa)
XP_035243482.1 Anguilla anguilla (1286 aa)
XP_018961588.1 Cyprinus carpio (1140 aa)
XP_026131542.1 Carassius auratus (1165 aa)
XP_021335504.1 Danio rerio (1230 aa)
XP_026884338.2 Electrophorus electricus (1195 aa)
XP_030632422.1 Chanos chanos (1133 aa)
XP_029631805.1 Salmo trutta (1228 aa)
XP_020559024.1 Oryzias latipes (1210 aa)
TDH13510.1 Perca flavescens (1144 aa)
XP_028432501.1 Perca flavescens (1073 aa)
XP_019734859.1 Hippocampus comes (1208 aa)
XP_030277208.1 Sparus aurata (1200 aa)
XP_031612859.1 Oreochromis aureus (1047 aa)
0.10

Supplementary Figure S5. Phylogenetic reconstruction of the vertebrate Plekha6 proteins. The evolutionary history of the vertebrate Plekha6 homologs was inferred using the Neighbor-Joining and the Maximum Likelihood methods as implemented in MEGA X. The percentage of replicate trees in which the associated taxa clustered together in the bootstrap test (1000 replicates) are shown next to the branches. In both methods the JTT matrix-based model was used. The optimal tree and the tree with the highest log likelihood (-16289.89) are shown. A discrete Gamma distribution was used to model evolutionary rate differences among sites (5 categories (+G, parameter = 1.1293)). All positions containing gaps and missing data were eliminated (complete deletion option). There was a total of 669 positions in the final dataset.

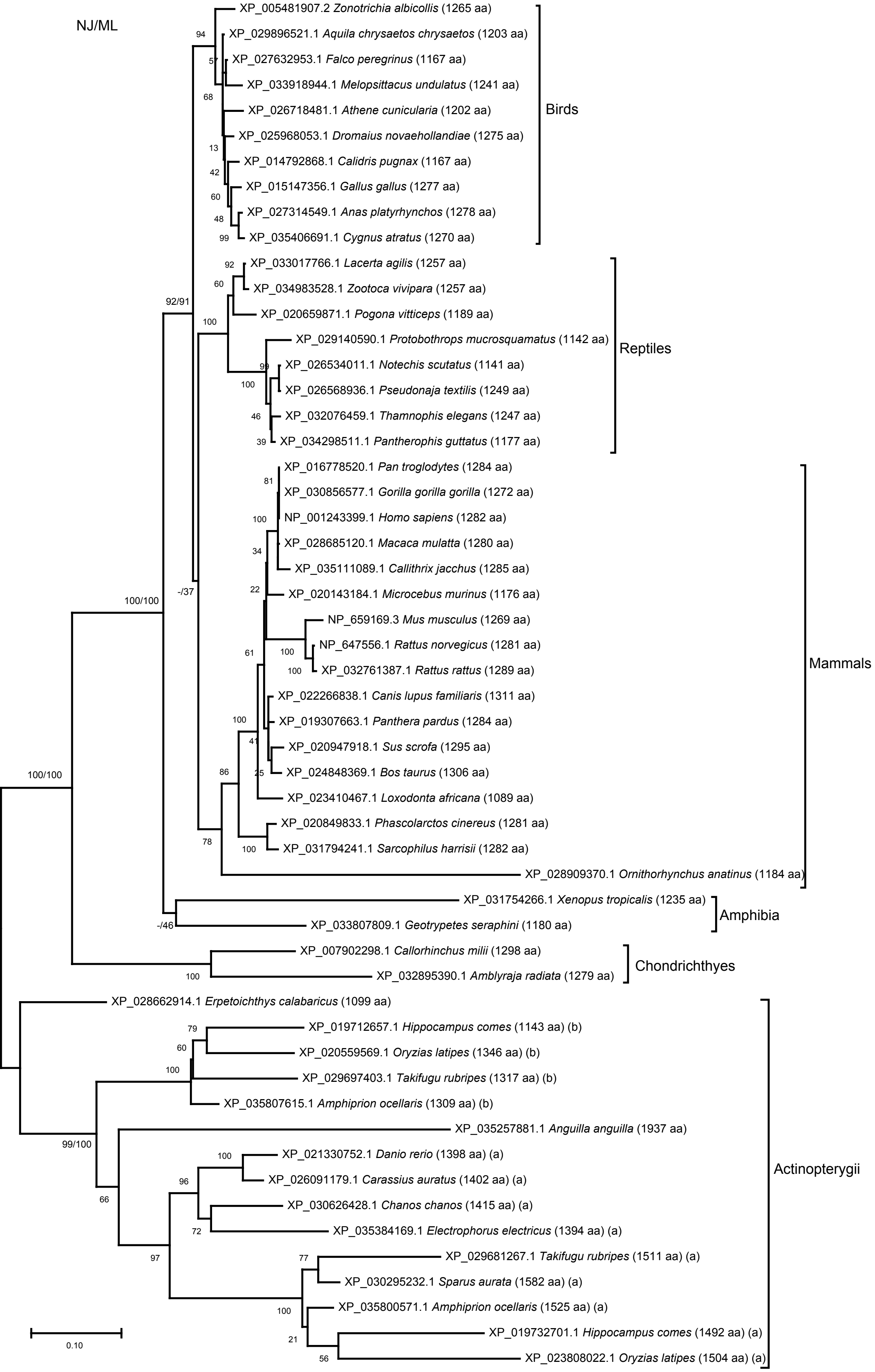

NJ/ML
XP_005481907.2 Zonotrichia albicollis (1265 aa)
XP_029896521.1 Aquila chrysaetos chrysaetos (1203 aa)
XP_027632953.1 Falco peregrinus (1167 aa)
XP_033918944.1 Melopsittacus undulatus (1241 aa)
XP_026718481.1 Athene cunicularia (1202 aa)
XP_025968053.1 Dromaius novaehollandiae (1275 aa)
XP_014792868.1 Calidris pugnax (1167 aa)
XP_015147356.1 Gallus gallus (1277 aa)
XP_027314549.1 Anas platyrhynchos (1278 aa)
XP_035406691.1 Cygnus atratus (1270 aa)
Birds
XP_033017766.1 Lacerta agilis (1257 aa)
XP_034983528.1 Zootoca vivipara (1257 aa)
XP_020659871.1 Pogona vitticeps (1189 aa)
XP_029140590.1 Protobothrops mucrosquamatus (1142 aa)
XP_026534011.1 Notechis scutatus (1141 aa)
XP_026568936.1 Pseudonaja textilis (1249 aa)
XP_032076459.1 Thamnophis elegans (1247 aa)
XP_034298511.1 Pantherophis guttatus (1177 aa)
Reptiles
XP_016778520.1 Pan troglodytes (1284 aa)
XP_030856577.1 Gorilla gorilla gorilla (1272 aa)
NP_001243399.1 Homo sapiens (1282 aa)
XP_028685120.1 Macaca mulatta (1280 aa)
XP_035111089.1 Callithrix jacchus (1285 aa)
XP_020143184.1 Microcebus murinus (1176 aa)
NP_659169.3 Mus musculus (1269 aa)
NP_647556.1 Rattus norvegicus (1281 aa)
XP_032761387.1 Rattus rattus (1289 aa)
XP_022266838.1 Canis lupus familiaris (1311 aa)
XP_019307663.1 Panthera pardus (1284 aa)
XP_020947918.1 Sus scrofa (1295 aa)
XP_024848369.1 Bos taurus (1306 aa)
XP_023410467.1 Loxodonta africana (1089 aa)
XP_020849833.1 Phascolarctos cinereus (1281 aa)
XP_031794241.1 Sarcophilus harrisii (1282 aa)
XP_028909370.1 Ornithorhynchus anatinus (1184 aa)
Mammals
XP_031754266.1 Xenopus tropicalis (1235 aa)
XP_033807809.1 Geotrypetes seraphini (1180 aa)
Amphibia
XP_007902298.1 Callorhinchus milii (1298 aa)
XP_032895390.1 Amblyraja radiata (1279 aa)
Chondrichthyes
XP_028662914.1 Erpetoichthys calabaricus (1099 aa)
XP_019712657.1 Hippocampus comes (1143 aa) (b)
XP_020559569.1 Oryzias latipes (1346 aa) (b)
XP_029697403.1 Takifugu rubripes (1317 aa) (b)
XP_035807615.1 Amphiprion ocellaris (1309 aa) (b)
XP_035257881.1 Anguilla anguilla (1937 aa)
XP_021330752.1 Danio rerio (1398 aa) (a)
XP_026091179.1 Carassius auratus (1402 aa) (a)
XP_030626428.1 Chanos chanos (1415 aa) (a)
XP_035384169.1 Electrophorus electricus (1394 aa) (a)
XP_029681267.1 Takifugu rubripes (1511 aa) (a)
XP_030295232.1 Sparus aurata (1582 aa) (a)
XP_035800571.1 Amphiprion ocellaris (1525 aa) (a)
XP_019732701.1 Hippocampus comes (1492 aa) (a)
XP_023808022.1 Oryzias latipes (1504 aa) (a)
Actinopterygii
94
57
68
13
42
60
48
99
92
60
100
99
100
46
39
92/91
81
100
34
22
100
100
61
41
25
100
86
100
78
-/37
100/100
-/46
100
100/100
79
60
100
99/100
100
96
72
66
97
77
100
21
56
0.10

Supplementary Figure S6. Phylogenetic reconstruction of the vertebrate Plekha5 proteins. The phylogenetic relationships between the Plekha5 proteins were inferred using the Neighbor-Joining and the Maximum Likelihood methods in MEGA X. The percentage of replicate trees in which the associated taxa clustered together in the bootstrap test (1000 replicates) are shown next to the branches. In both methods the JTT matrix-based model was used. The optimal tree and the tree with the highest log likelihood (-13844.45) are shown. A discrete Gamma distribution was used to model evolutionary rate differences among sites (5 categories (+G, parameter = 1.0091)). All positions containing gaps and missing data were eliminated (complete deletion option). There was a total of 646 positions in the final dataset. Teleost-specific copies are indicated by a and b.

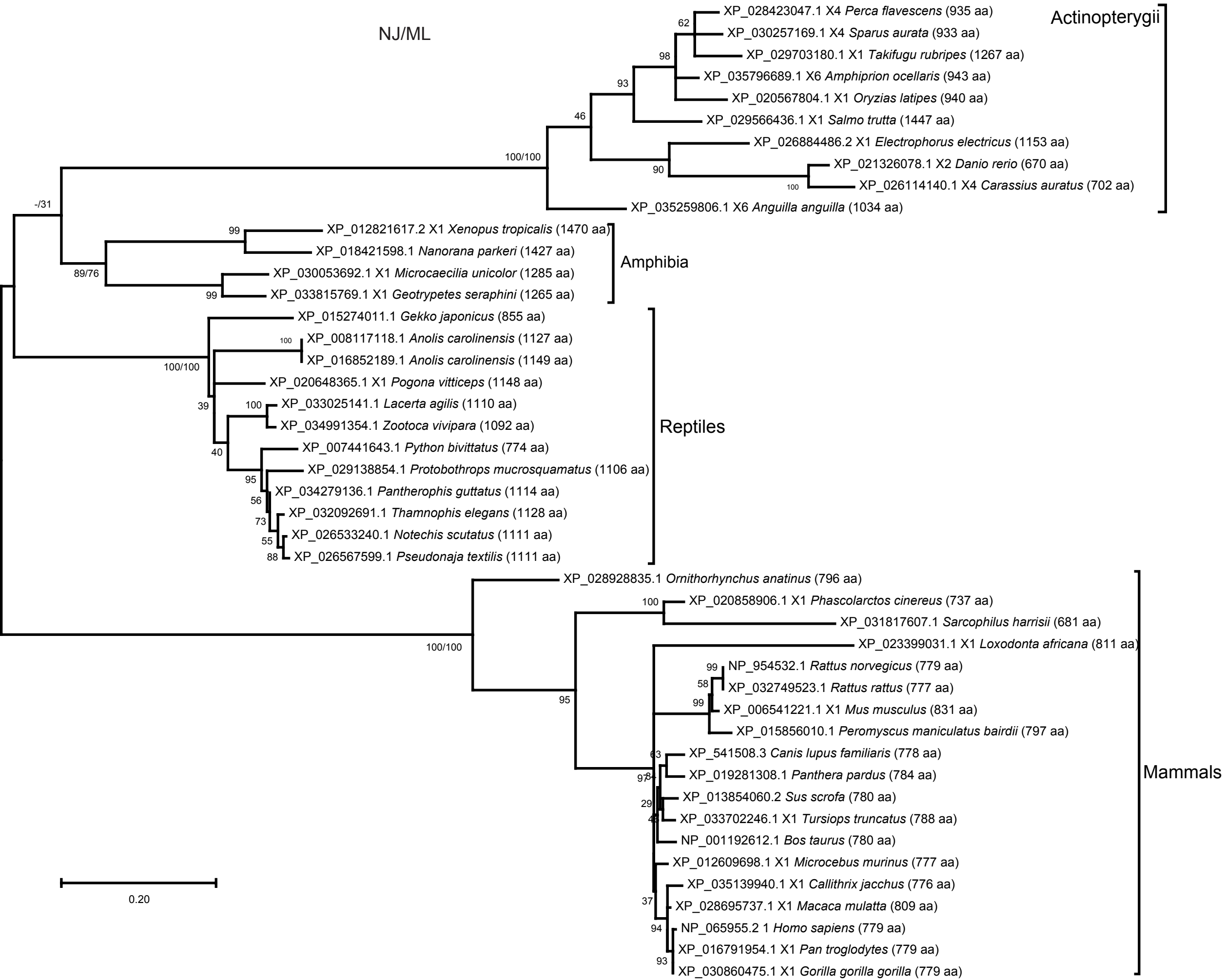
NJ/ML
XP_028423047.1 X4 Perca flavescens (935 aa)
XP_030257169.1 X4 Sparus aurata (933 aa)
XP_029703180.1 X1 Takifugu rubripes (1267 aa)
XP_035796689.1 X6 Amphiprion ocellaris (943 aa)
XP_020567804.1 X1 Oryzias latipes (940 aa)
XP_029566436.1 X1 Salmo trutta (1447 aa)
XP_026884486.2 X1 Electrophorus electricus (1153 aa)
XP_021326078.1 X2 Danio rerio (670 aa)
XP_026114140.1 X4 Carassius auratus (702 aa)
XP_035259806.1 X6 Anguilla anguilla (1034 aa)
Actinopterygii
XP_012821617.2 X1 Xenopus tropicalis (1470 aa)
XP_018421598.1 Nanorana parkeri (1427 aa)
XP_030053692.1 X1 Microcaecilia unicolor (1285 aa)
XP_033815769.1 X1 Geotrypetes seraphini (1265 aa)
Amphibia
XP_015274011.1 Gekko japonicus (855 aa)
XP_008117118.1 Anolis carolinensis (1127 aa)
XP_016852189.1 Anolis carolinensis (1149 aa)
XP_020648365.1 X1 Pogona vitticeps (1148 aa)
XP_033025141.1 Lacerta agilis (1110 aa)
XP_034991354.1 Zootoca vivipara (1092 aa)
XP_007441643.1 Python bivittatus (774 aa)
XP_029138854.1 Protobothrops mucrosquamatus (1106 aa)
XP_034279136.1 Pantherophis guttatus (1114 aa)
XP_032092691.1 Thamnophis elegans (1128 aa)
XP_026533240.1 Notechis scutatus (1111 aa)
XP_026567599.1 Pseudonaja textilis (1111 aa)
Reptiles
XP_028928835.1 Ornithorhynchus anatinus (796 aa)
XP_020858906.1 X1 Phascolarctos cinereus (737 aa)
XP_031817607.1 Sarcophilus harrisii (681 aa)
XP_023399031.1 X1 Loxodonta africana (811 aa)
NP_954532.1 Rattus norvegicus (779 aa)
XP_032749523.1 Rattus rattus (777 aa)
XP_006541221.1 X1 Mus musculus (831 aa)
XP_015856010.1 Peromyscus maniculatus bairdii (797 aa)
XP_541508.3 Canis lupus familiaris (778 aa)
XP_019281308.1 Panthera pardus (784 aa)
XP_013854060.2 Sus scrofa (780 aa)
XP_033702246.1 X1 Tursiops truncatus (788 aa)
NP_001192612.1 Bos taurus (780 aa)
XP_012609698.1 X1 Microcebus murinus (777 aa)
XP_035139940.1 X1 Callithrix jacchus (776 aa)
XP_028695737.1 X1 Macaca mulatta (809 aa)
NP_065955.2 1 Homo sapiens (779 aa)
XP_016791954.1 X1 Pan troglodytes (779 aa)
XP_030860475.1 X1 Gorilla gorilla gorilla (779 aa)
Mammals
0.20

Supplementary Figure S7. Phylogenetic reconstruction of the vertebrate Plekha4 proteins. The evolutionary relationships between the Plekha4 homologs were inferred using the Neighbor-Joining and the Maximum Likelihood methods. The percentage of replicate trees in which the associated taxa clustered together in the bootstrap test (1000 replicates) are shown next to the branches. In both methods the JTT matrix-based model was used. The optimal tree and the tree with the highest log likelihood (-6607.77) are shown. A discrete Gamma distribution was used to model evolutionary rate differences among sites (5 categories (+G, parameter = 1.4245)). The rate variation model allowed for some sites to be evolutionarily invariable ([+I], 8.66% sites). All positions containing gaps and missing data were eliminated (complete deletion option). There was a total of 583 positions in the final dataset.

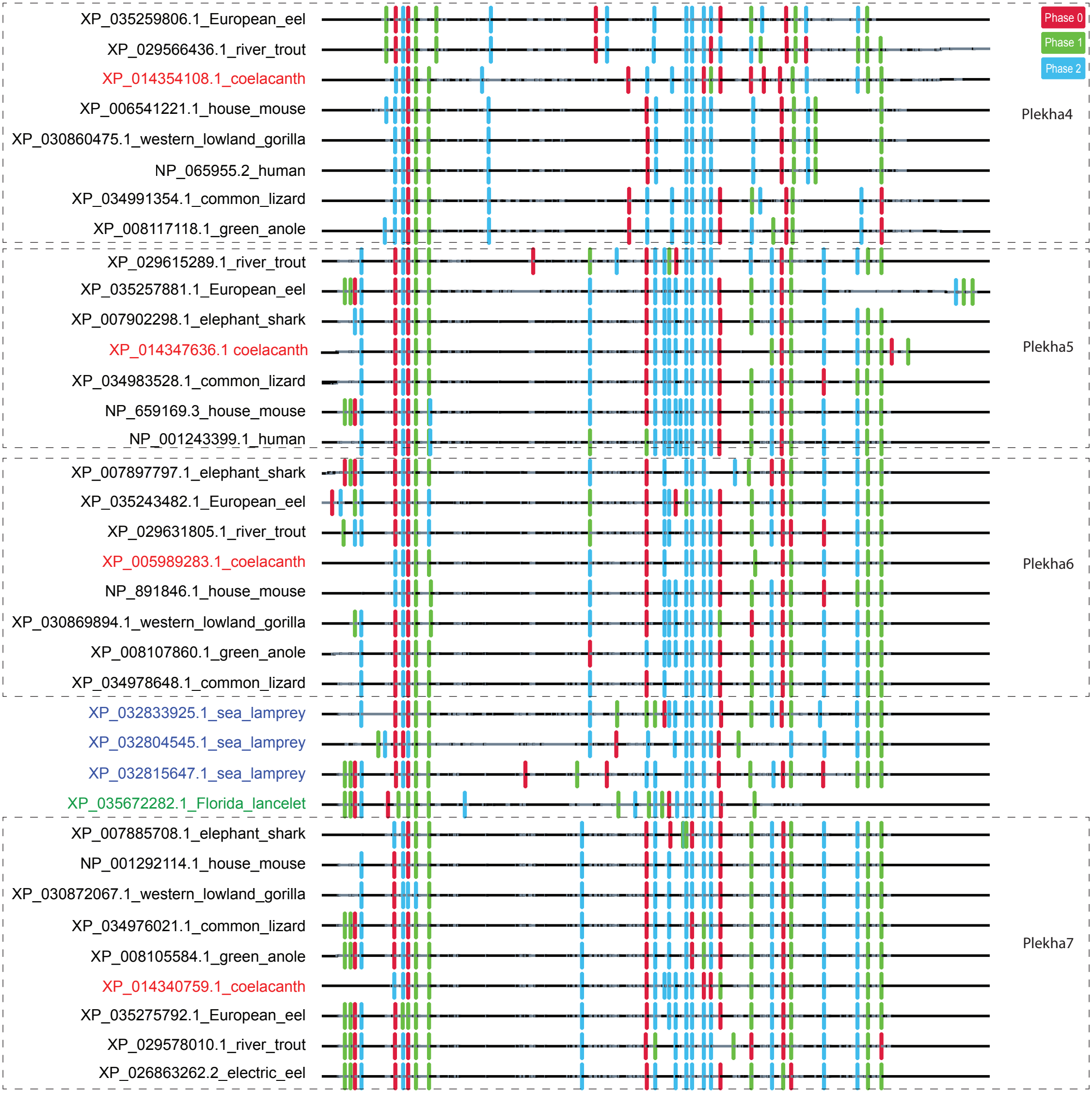
Phase 0
Phase 1
Phase 2
XP_035259806.1_European_eel
XP_029566436.1_river_trout
XP_014354108.1_coelacanth
XP_006541221.1_house_mouse
XP_030860475.1_western_lowland_gorilla
NP_065955.2_human
XP_034991354.1_common_lizard
XP_008117118.1_green_anole
Plekha4
XP_029615289.1_river_trout
XP_035257881.1_European_eel
XP_007902298.1_elephant_shark
XP_014347636.1 coelacanth
XP_034983528.1_common_lizard
NP_659169.3_house_mouse
NP_001243399.1_human
Plekha5
XP_007897797.1_elephant_shark
XP_035243482.1_European_eel
XP_029631805.1_river_trout
XP_005989283.1_coelacanth
NP_891846.1_house_mouse
XP_030869894.1_western_lowland_gorilla
XP_008107860.1_green_anole
XP_034978648.1_common_lizard
Plekha6
XP_032833925.1_sea_lamprey
XP_032804545.1_sea_lamprey
XP_032815647.1_sea_lamprey
XP_035672282.1_Florida_lancelet
XP_007885708.1_elephant_shark
NP_001292114.1_house_mouse
XP_030872067.1_western_lowland_gorilla
XP_034976021.1_common_lizard
XP_008105584.1_green_anole
XP_014340759.1_coelacanth
XP_035275792.1_European_eel
XP_029578010.1_river_trout
XP_026863262.2_electric_eel
Plekha7

Supplementary Figure S8. Conservation of intron position and phase of the different chordate *Plekha4,5,6,7* genes. The figure depicts the position and phase of introns within *Plekha4,5,6,7* genes from representative species. The intron positions were mapped on a multiple sequence alignment and denoted with colored boxes (phase 0: red; phase 1: green; phase 2: blue). The parts of the multiple sequence alignment that contain information (amino acids) are shown as black lines and the ones containing alignment gaps are shown are light grey lines. The intron collection, mapping, and coloring were performed using *in-house* scripts (see Methods).

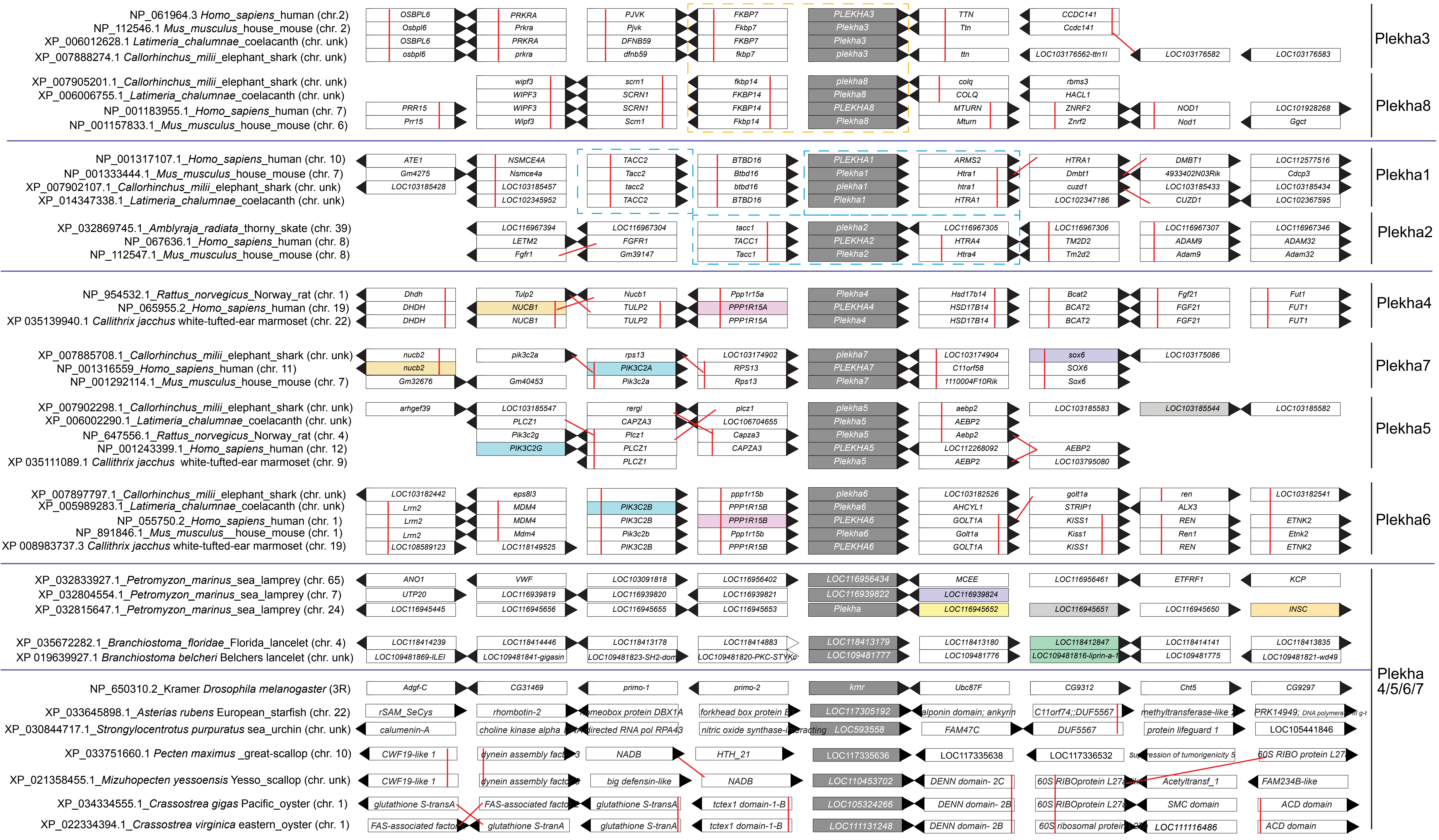

NP_061964.3 Homo_sapiens_human (chr.2)
NP_112546.1 Mus_musculus_house_mouse (chr. 2)
XP_006012628.1 Latimeria_chalumnae_coelacanth (chr. unk)
XP_007888274.1 Callorhinchus_milii_elephant_shark (chr. unk)
OSBPL6 Osbpl6 OSBPL6 osbpl6
PRKRA Prkra PRKRA prkra
PJVK Pjvk DFNB59 dfnb59
FKBP7 Fkbp7 FKBP7 fkbp7
PLEKHA3 Plekha3 Plekha3 plekha3
TTN Ttn ttn
CCDC141 Ccdc141 LOC103176562-ttn1l
LOC103176582
LOC103176583
Plekha3
XP_007905201.1_Callorhinchus_milii_elephant_shark (chr. unk)
XP_006006755.1_Latimeria_chalumnae_coelacanth (chr. unk)
NP_001183955.1_Homo_sapiens_human (chr. 7)
NP_001157833.1_Mus_musculus_house_mouse (chr. 6)
PRR15 Prr15
wipf3 WIPF3 WIPF3 Wipf3
scrn1 SCRN1 SCRN1 Scrn1
fkbp14 FKBP14 FKBP14 Fkbp14
plekha8 Plekha8 PLEKHA8 Plekha8
colq COLQ MTURN Mturn
rbms3 HACL1 ZNRF2 Znrf2
NOD1 Nod1
LOC101928268 Ggct
Plekha8
NP_001317107.1_Homo_sapiens_human (chr. 10)
NP_001333444.1_Mus_musculus_house_mouse (chr. 7)
XP_007902107.1_Callorhinchus_milii_elephant_shark (chr. unk)
XP_014347338.1_Latimeria_chalumnae_coelacanth (chr. unk)
ATE1 Gm4275 LOC103185428
NSMCE4A Nsmce4a LOC103185457 LOC102345952
TACC2 Tacc2 tacc2 TACC2
BTBD16 Btbd16 btbd16 BTBD16
PLEKHA1 Plekha1 plekha1 Plekha1
ARMS2 Htra1 htra1 HTRA1
HTRA1 Dmbt1 cuzd1 LOC102347186
DMBT1 4933402N03Rik LOC103185433 CUZD1
LOC112577516 Cdcp3 LOC103185434 LOC102367595
Plekha1
XP_032869745.1_Amblyraja_radiata_thorny_skate (chr. 39)
NP_067636.1_Homo_sapiens_human (chr. 8)
NP_112547.1_Mus_musculus_house_mouse (chr. 8)
LOC116967394 LETM2 Fgfr1
LOC116967304 FGFR1 Gm39147
tacc1 TACC1 Tacc1
plekha2 PLEKHA2 Plekha2
LOC116967305 HTRA4 Htra4
LOC116967306 TM2D2 Tm2d2
LOC116967307 ADAM9 Adam9
LOC116967346 ADAM32 Adam32
Plekha2
NP_954532.1_Rattus_norvegicus_Norway_rat (chr. 1)
NP_065955.2_Homo_sapiens_human (chr. 19)
XP 035139940.1 Callithrix jacchus white-tufted-ear marmoset (chr. 22)
Dhdh DHDH DHDH
Tulp2 NUCB1 NUCB1
Nucb1 TULP2 TULP2
Ppp1r15a PPP1R15A PPP1R15A
Plekha4 PLEKHA4 Plekha4
Hsd17b14 HSD17B14 HSD17B14
Bcat2 BCAT2 BCAT2
Fgf21 FGF21 FGF21
Fut1 FUT1 FUT1
Plekha4
XP_007885708.1_Callorhinchus_milii_elephant_shark (chr. unk)
NP_001316559_Homo_sapiens_human (chr. 11)
NP_001292114.1_Mus_musculus_house_mouse (chr. 7)
nucb2 nucb2 Gm32676
pik3c2a Gm40453
rps13 PIK3C2A Pik3c2a
LOC103174902 RPS13 Rps13
plekha7 PLEKHA7 Plekha7
LOC103174904 C11orf58 1110004F10Rik
sox6 SOX6 Sox6
LOC103175086
Plekha7
XP_007902298.1_Callorhinchus_milii_elephant_shark (chr. unk)
XP_006002290.1_Latimeria_chalumnae_coelacanth (chr. unk)
NP_647556.1_Rattus_norvegicus_Norway_rat (chr. 4)
NP_001243399.1_Homo_sapiens_human (chr. 12)
XP 035111089.1 Callithrix jacchus white-tufted-ear marmoset (chr. 9)
arhgef39
LOC103185547 PLCZ1 Pik3c2g PIK3C2G
rergl CAPZA3 Plcz1 PLCZ1 PLCZ1
plcz1 LOC106704655 Capza3 CAPZA3
plekha5 Plekha5 Plekha5 PLEKHA5 Plekha5
aebp2 AEBP2 Aebp2 LOC112268092 AEBP2
LOC103185583 AEBP2 LOC103795080
LOC103185544
LOC103185582
Plekha5
XP_007897797.1_Callorhinchus_milii_elephant_shark (chr. unk)
XP_005989283.1_Latimeria_chalumnae_coelacanth (chr. unk)
NP_055750.2_Homo_sapiens_human (chr. 1)
NP_891846.1_Mus_musculus_house_mouse (chr. 1)
XP 008983737.3 Callithrix jacchus white-tufted-ear marmoset (chr. 19)
LOC103182442 Lrrn2 Lrrn2 Lrrn2 LOC108589123
eps8l3 MDM4 MDM4 Mdm4 LOC118149525
PIK3C2B PIK3C2B Pik3c2b PIK3C2B
ppp1r15b PPP1R15B PPP1R15B Ppp1r15b PPP1R15B
plekha6 Plekha6 PLEKHA6 Plekha6 PLEKHA6
LOC103182526 AHCYL1 GOLT1A Golt1a GOLT1A
golt1a STRIP1 KISS1 Kiss1 KISS1
ren ALX3 REN Ren1 REN
LOC103182541 ETNK2 Etnk2 ETNK2
Plekha6
XP_032833927.1_Petromyzon_marinus_sea_lamprey (chr. 65)
XP_032804554.1_Petromyzon_marinus_sea_lamprey (chr. 7)
XP_032815647.1_Petromyzon_marinus_sea_lamprey (chr. 24)
ANO1 UTP20 LOC116945445
VWF LOC116939819 LOC116945656
LOC103091818 LOC116939820 LOC116945655
LOC116956402 LOC116939821 LOC116945653
LOC116956434 LOC116939822 Plekha
MCEE LOC116939824 LOC116945652
LOC116956461 LOC116945651
ETFRF1 LOC116945650
KCP INSC
XP_035672282.1_Branchiostoma_floridae_Florida_lancelet (chr. 4)
XP 019639927.1 Branchiostoma belcheri Belchers lancelet (chr. unk)
LOC118414239 LOC109481869-ILEI
LOC118414446 LOC109481841-gigasin
LOC118413178 LOC109481823-SH2-dom
LOC118414883 LOC109481820-PKC-STYKc
LOC118413179 LOC109481777
LOC118413180 LOC109481776
LOC118412847 LOC109481816-liprin-a-1
LOC118414141 LOC109481775
LOC118413835 LOC109481821-wd49
NP_650310.2_Kramer Drosophila melanogaster (3R)
Adgf-C CG31469 primo-1 primo-2 kmr Ubc87F CG9312 Cht5 CG9297
XP_033645898.1_Asterias rubens European_starfish (chr. 22)
XP_030844717.1_Strongylocentrotus purpuratus sea_urchin (chr. unk)
rSAM_SeCys rhombotin-2 homeobox protein DBX1A forkhead box protein B LOC117305192 calponin domain; ankyrin C1orf74;;DUF5567 methyltransferase-like 27 PRK14949; DNA polymerase III g-t
calumenin-A choline kinase alpha DNA-directed RNA pol RPA43 nitric oxide synthase-interacting LOC593558 FAM47C DUF5567 protein lifeguard 1 LOC105441846
XP_033751660.1 Pecten maximus _great-scallop (chr. 10)
XP_021358455.1_Mizuhopecten yessoensis Yesso_scallop (chr. unk)
CWF19-like 1 dynein assembly factor 3 NADB HTH_21 LOC117335636 LOC117335638 LOC117336532 suppression of tumorigenicity 5 60S RIBO protein L27a
CWF19-like 1 dynein assembly factor 3 big defensin-like NADB LOC110453702 DENN domain- 2C 60S RIBOprotein L27a-like Acetyltransf_1 FAM234B-like
XP_034334555.1_Crassostrea gigas Pacific_oyster (chr. 1)
XP_022334394.1_Crassostrea virginica eastern_oyster (chr. 1)
glutathione S-transA FAS-associated factor 2 glutathione S-transA tctex1 domain-1-B LOC105324266 DENN domain- 2B 60S RIBOprotein L27a SMC domain ACD domain
FAS-associated factor 2 glutathione S-tranA glutathione S-tranA tctex1 domain-1-B LOC111131248 DENN domain- 2B 60S ribosomal protein L27a LOC111116486 ACD domain
Plekha 4/5/6/7

Supplementary Figure S9. Genomic organization of the *Plekha1-8* loci further depicts three distinct subfamilies and the distinct origins of the *PLEKHA4/5/6/7* subfamily. The first four protein coding genes and up to 500 kbps flanking representative *Plekha4,5,6,7* loci were collected from GenBank using *in-house* scripts (see Methods). The gene name and domain information for each locus were collected and analyzed to suggest homology and reveal conserved synteny. Pairwise alignments were used as needed to supplement the above analyses. Genes are represented as arrows and each arrow's direction represents the gene's transcription orientation. Red lines are used to connect orthologous genes. Genes are colored only if they were shared at least between two paralogous gene groups. The graph is not in scale for clarity.

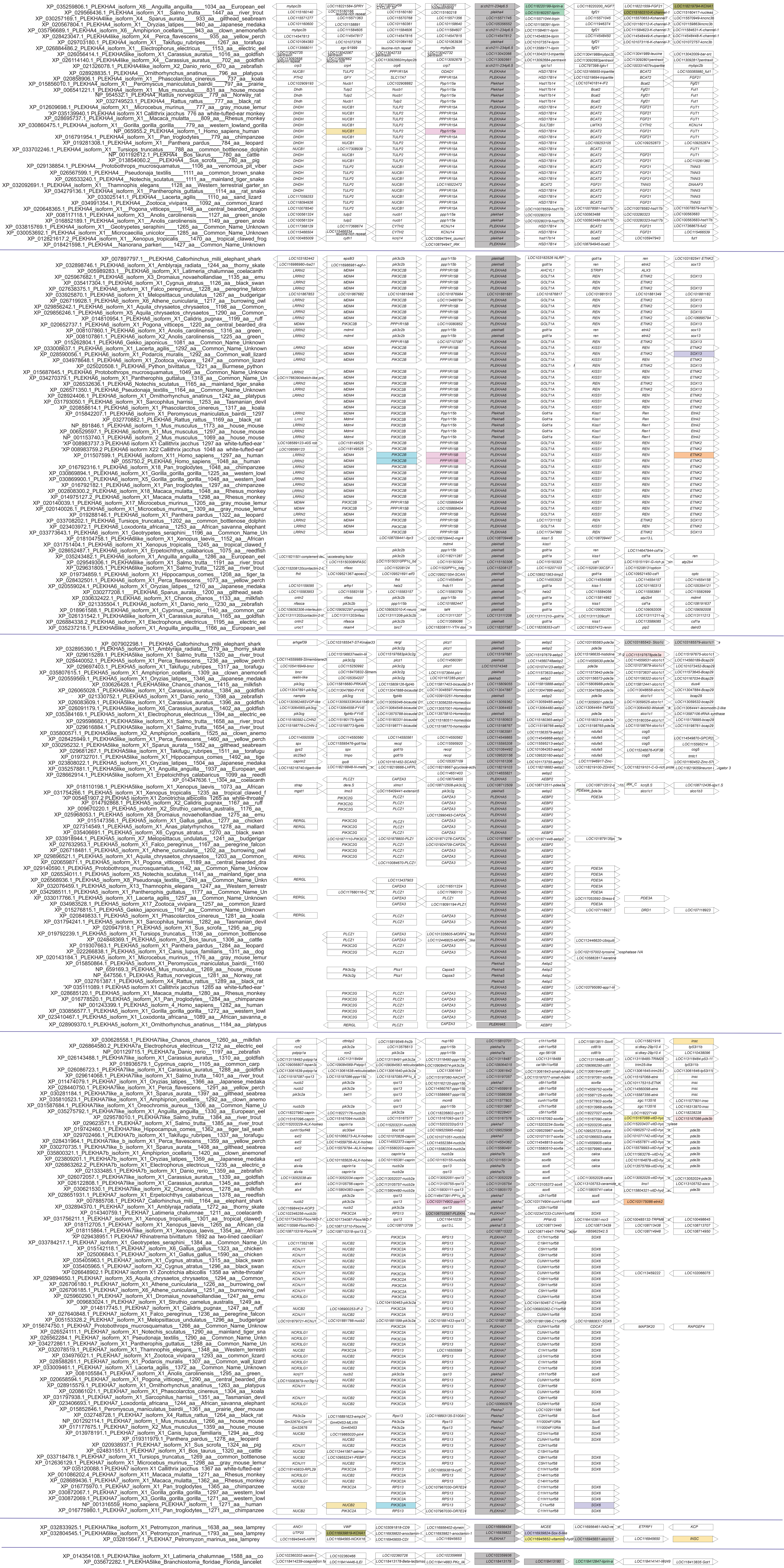

Supplementary Figure S10. Genomic organization of the *PLEKHA4/5/6/7* subfamily. The first four protein coding genes and up to 500 kbps flanking representative *Plekha4,5,6,7* loci were collected from GenBank using *in-house* scripts (see Methods). The gene name and domain information for each locus were collected and analyzed to suggest homology and reveal conserved synteny. Pairwise alignments were used as needed to supplement the above analyses. Genes are represented as arrows and each arrow's direction represents the gene's transcription orientation. Genes are colored only if they were shared at least between two paralogous gene groups. The graph is not in scale for clarity. This figure shows the complete dataset of the representative sample shown in **Figure 4**.

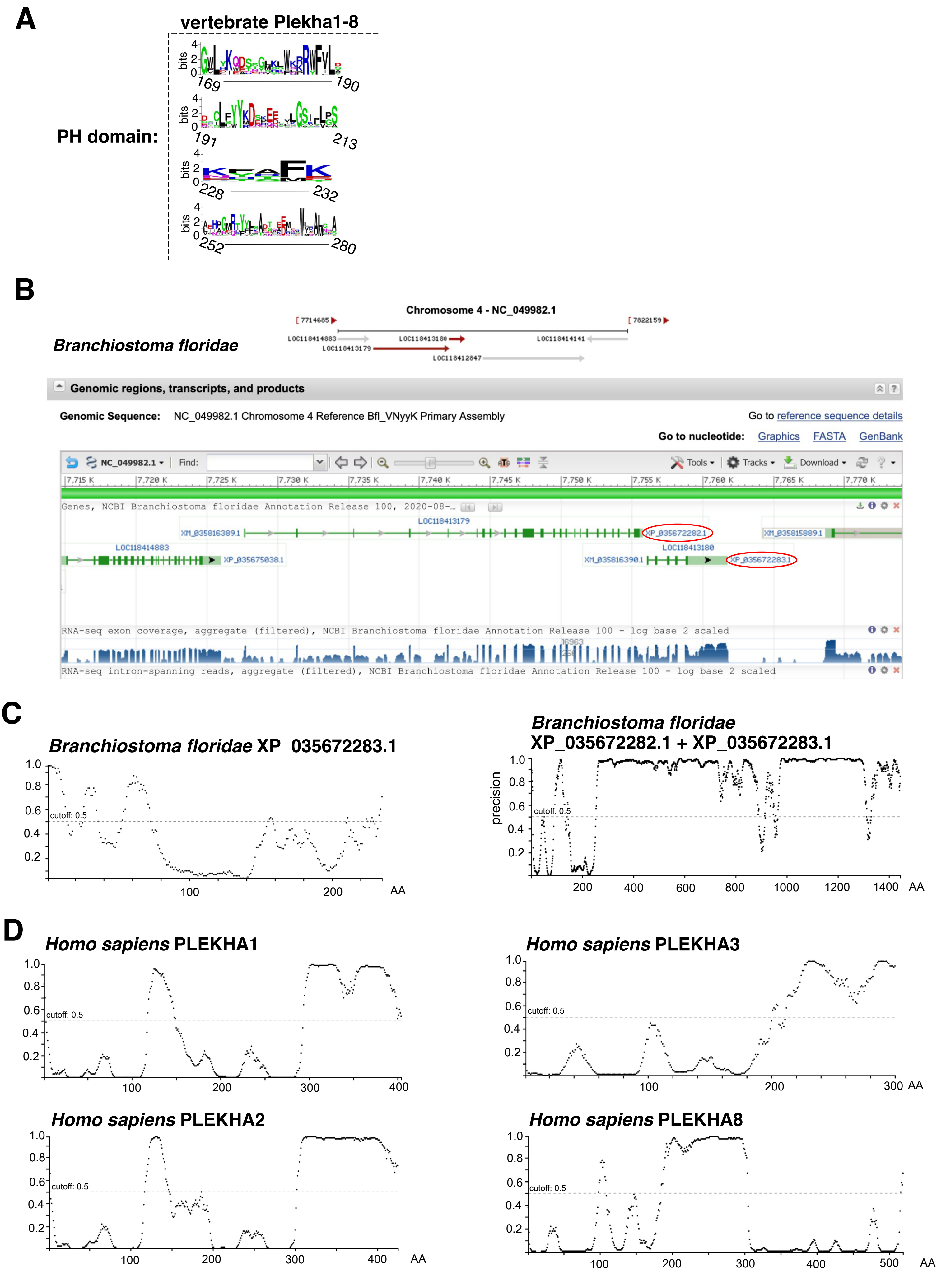

A
vertebrate Plekha1-8
PH domain:
bits
169
190
191
213
228
232
252
280
B
Chromosome 4 - NC_049982.1
7714685
7822159
Branchiostoma floridae
LOC118414883
LOC118413180
LOC118414141
LOC118413179
LOC118412847
Genomic regions, transcripts, and products
Genomic Sequence: NC_049982.1 Chromosome 4 Reference Bfl_VNyyK Primary Assembly
Go to reference sequence details
Go to nucleotide: Graphics FASTA GenBank
NC_049982.1
Find:
Tools
Tracks
Download
7,715 K
7,720 K
7,725 K
7,730 K
7,735 K
7,740 K
7,745 K
7,750 K
7,755 K
7,760 K
7,765 K
7,770 K
Genes, NCBI Branchiostoma floridae Annotation Release 100, 2020-08-...
XM_035816389.1
XP_035672282.1
XM_035815889.1
XP_035675038.1
XM_035816390.1
XP_035672283.1
LOC118413180
RNA-seq exon coverage, aggregate (filtered), NCBI Branchiostoma floridae Annotation Release 100 - log base 2 scaled
RNA-seq intron-spanning reads, aggregate (filtered), NCBI Branchiostoma floridae Annotation Release 100 - log base 2 scaled
C
Branchiostoma floridae XP_035672283.1
Branchiostoma floridae
XP_035672282.1 + XP_035672283.1
precision
cutoff: 0.5
AA
D
Homo sapiens PLEKHA1
Homo sapiens PLEKHA3
Homo sapiens PLEKHA2
Homo sapiens PLEKHA8

Supplementary Figure S11. (A) Sequence conservation analysis of the PH domain of the vertebrate Plekha1-8 proteins, as determined by WebLogo analysis of the multiple sequence alignments used for the phylogenetic analysis of **Figure 2A**. (B) Snapshot of the *Branchiostoma floridae* genomic region containing the tandem XP_035672282.1 and XP_035672283.1 open reading frames. (C) Intrinsically disorder domain prediction analysis of the *Branchiostoma floridae* XP_035672283.1 protein fragment (left), as well as of the XP_035672282.1 and XP_035672283.1, when artificially merged into one protein sequence (right). (D) Intrinsically disorder domain prediction analysis of the human PLEKHA1, 2, 3, 8 proteins.
